# Association between insulin resistance-related indices and cardiovascular disease in patients with atopic dermatitis: mutual validation using real-world and NHANES data

**DOI:** 10.3389/fcvm.2026.1903179

**Published:** 2026-09-29

**Authors:** Yu An, Han Zhou, Liangjuan Ma

**Affiliations:** 1 Department of Dermatology, the First Affiliated Hospital of Harbin Medical University, Harbin, Heilongjiang, China; 2 Department of Cardiology, the First Affiliated Hospital of Harbin Medical University, Harbin, Heilongjiang, China

**Keywords:** atopic dermatitis, cardiovascular disease, insulin resistance, machine learning, TyG

## Abstract

**Background:**

The number of patients with atopic dermatitis (AD) who develop cardiovascular disease (CVD) has been increasing year by year. However, reliable biomarkers for identifying AD patients with a higher burden of prevalent CVD remain lacking.

**Methods:**

This study included patient data from the First Affiliated Hospital of Harbin Medical University from 2014 to 2023 and data from the U.S. National Health and Nutrition Examination Survey (NHANES). Multivariable logistic regression analyses were performed to evaluate the associations of triglyceride-glucose index (TyG), TyG-body mass index (TyG-BMI), C-reactive protein–triglyceride-glucose index (CTI), and CTI-body mass index (CTI-BMI) with prevalent CVD in patients with AD. Restricted cubic spline (RCS) analyses, subgroup analyses, and sensitivity analyses were conducted to evaluate the robustness of the findings. Seven machine learning (ML) models and SHAP analysis were applied to assess the discriminative performance of these indices for prevalent CVD status.

**Results:**

In the real-world dataset, 145 patients had prevalent CVD, whereas 75 participants had prevalent CVD in the NHANES dataset. Multivariable logistic regression analyses showed that, after adjustment for clinical covariates, all four indices were significantly and positively associated with prevalent CVD among patients with AD. These associations remained consistent across most subgroup analyses. Restricted cubic spline analyses showed no evidence of nonlinear associations, and sensitivity analyses supported the robustness of the findings. Among the evaluated ML models, CTI-BMI demonstrated the highest discriminative performance for prevalent CVD status in both datasets and contributed most substantially to model discrimination.

**Conclusion:**

This study demonstrated that TyG, TyG-BMI, CTI, and CTI-BMI were significantly associated with prevalent CVD among patients with AD. CTI-BMI showed the strongest discriminative performance among the evaluated indices and provided modest incremental discrimination when incorporated into machine learning models. These findings suggest that these indices may serve as potential adjunctive markers for cardiovascular burden assessment in patients with AD; however, further prospective studies are required to determine their value for predicting future cardiovascular events.

## Graphical Abstract

**Figure:**
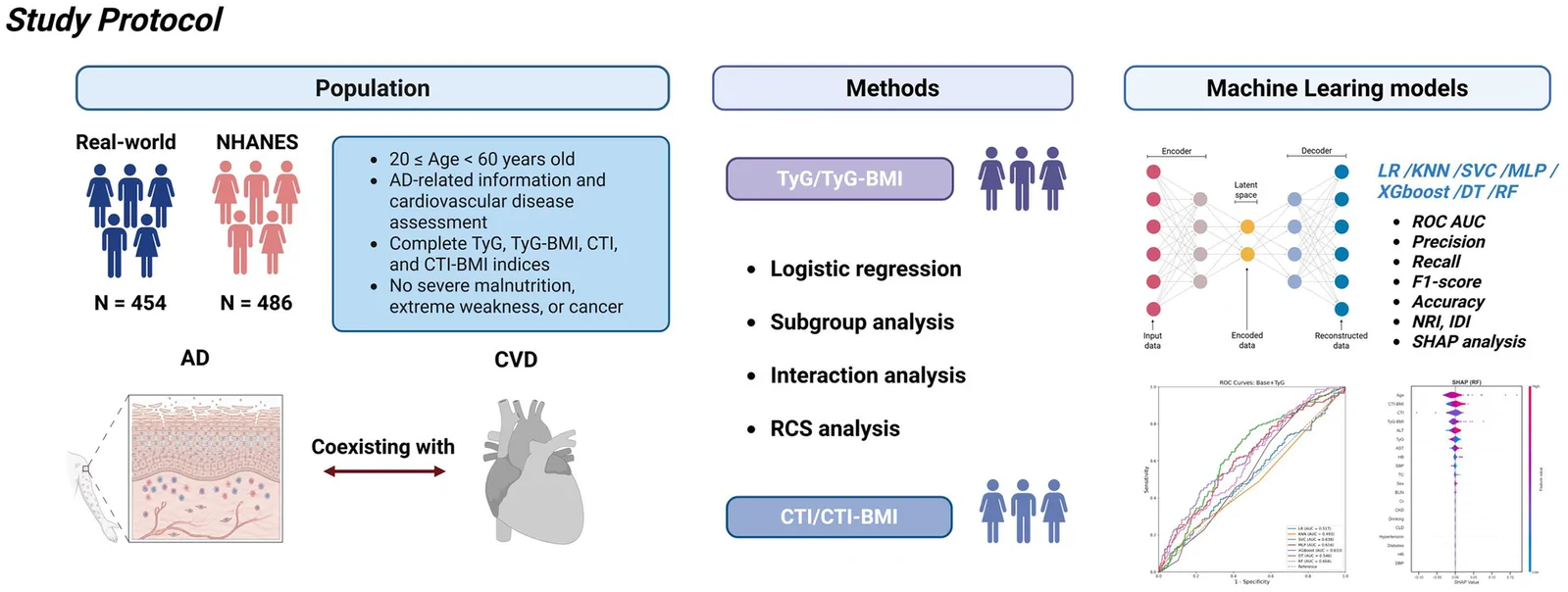

## 1 Introduction

Atopic dermatitis (AD) is a chronic and recurrent inflammatory skin disease, with an estimated prevalence of approximately 5%–10% among adults and a gradually increasing trend over time (1, 2). AD is closely associated with immune dysregulation and impaired skin barrier function, and is often accompanied by systemic inflammatory manifestations (3). Therefore, in addition to local cutaneous symptoms such as intense pruritus, skin dryness, and eczematous lesions, AD is frequently comorbid with other allergic diseases, including allergic rhinitis and asthma (4, 5). Existing evidence also suggests that AD is closely associated with cardiovascular disease (CVD) (6). Individuals with severe and persistent AD have an increased susceptibility to CVD (7). This association is considered to be related to systemic inflammatory responses and metabolic disorders (8).

A study of 34,525 adults aged 18–85 years in the United States found that adults with AD had more cardiovascular risk factors than those without AD (9). In addition, a meta-analysis reported that AD was associated with a higher prevalence of cardiovascular comorbidities, including myocardial infarction (MI), stroke, angina, and heart failure, and that greater AD severity was associated with a higher cardiovascular burden (10). Globally, CVD-related deaths account for approximately 30% of all deaths, representing a substantial disease burden (11). In recent years, researchers have increasingly focused on identifying biomarkers for assessing CVD risk in patients with AD.

Insulin resistance (IR) is an important risk factor for metabolic dysfunction and CVD (12). IR is defined as a state of reduced insulin sensitivity and responsiveness, which can lead to impaired glucose uptake, hyperglycemia, and various metabolic disorders (13). IR may increase the risk of CVD through several mechanisms, including excessive activation of the sympathetic nervous system, impaired vascular endothelial function, and inflammation (14, 15). Several composite metabolic indices incorporating glucose, lipid, inflammatory, and adiposity-related parameters have been proposed as surrogate indicators associated with insulin resistance, including the triglyceride-glucose (TyG) index, TyG-body mass index (TyG-BMI), C-reactive protein–TyG index (CTI), and CTI-body mass index (CTI-BMI) (16, 17). However, whether these indices accurately reflect insulin resistance specifically in patients with AD remains insufficiently established. Multiple observational studies have shown that elevated TyG, TyG-BMI, CTI, and CTI-BMI indices are associated with prevalent CVD or cardiovascular burden in the general population (18–20). However, the association between these indices and prevalent CVD among patients with AD remains unclear.

This study analyzed real-world data from the First Affiliated Hospital of Harbin Medical University and data from the U.S. National Health and Nutrition Examination Survey (NHANES), with reciprocal validation between the two datasets. The aim was to investigate the associations between TyG, TyG-BMI, CTI, and CTI-BMI indices and prevalent CVD among patients with AD, and to evaluate the discriminative performance of machine learning models incorporating these indices. SHAP analysis was further performed to assess the contribution of these indices to model discrimination. By evaluating the relationship between metabolic-inflammatory indices and prevalent CVD status in patients with AD, this study may provide insights into cardiovascular burden assessment and help identify patients who may benefit from comprehensive cardiovascular evaluation.

## 2 Methods

### 2.1 Data

The NHANES dataset has been described in detail in previous studies (21). Information on eczema was available in the 2001–2006 NHANES cycles. In this cross-sectional study, de-identified data from participants aged 20–59 years were extracted from the 2001–2006 NHANES cycles. All procedures were conducted in accordance with the guidelines approved by the Research Ethics Review Board of the National Center for Health Statistics. The inclusion criteria were as follows: (1) 20≤ age <60 years; (2) availability of AD-related information and cardiovascular disease assessment; (3) availability of essential laboratory measurements required for calculation of TyG, TyG-BMI, CTI, and CTI-BMI indices. Participants were excluded if they met any of the following criteria: (1) missing essential information required for defining AD status or CVD outcome; (2) missing fasting glucose, triglyceride, or CRP measurements required for index calculation; (3) presence of severe malnutrition, extreme frailty, or cancer. A total of 486 participants were included in this study.

In NHANES, eczema status was defined based on self-reported physician-diagnosed eczema, which was used as a proxy measure for AD-related conditions but does not represent dermatologist-confirmed AD.

In addition, this study included clinical data from the First Affiliated Hospital of Harbin Medical University between 2014 and 2023. This study was approved by the Ethics Review Committee of the First Affiliated Hospital of Harbin Medical University (approval date: June 25, 2024; ethics approval No. 20240258). Given the retrospective nature of this study and the use of de-identified clinical data, the requirement for written informed consent was waived by the ethics committee. In the real-world dataset, AD was diagnosed by two or more dermatologists according to clinical criteria. The inclusion and exclusion criteria were the same as those used for the NHANES dataset. A total of 454 patients were included in this study.

### 2.2 Definitions of TyG, TyG-BMI, CTI, and CTI-BMI indices and assessment of AD

The indices were defined as follows: TyG: ln [TG (mg/dL) × FBG (mg/dL)/2]; TyG-BMI: TyG × BMI; CTI: TyG + 0.412 × ln [CRP (mg/L)]; CTI-BMI: CTI × BMI.

In NHANES, AD was assessed based on the question, “Has a doctor or other health professional ever told you that you had eczema?” The corresponding variables were DED061 in the NHANES 2001–2004 cycles and AGQ180 in the NHANES 2005–2006 cycle (22). In the real-world dataset, the diagnosis of AD was jointly determined by two or more specialist physicians.

### 2.3 Outcome

The primary outcome of this study was cardiovascular disease, including coronary heart disease, heart failure, acute myocardial infarction, atrial fibrillation, stroke, and cardiac death. In NHANES, CVD was identified based on self-reported physician diagnoses obtained through standardized medical condition questionnaires during personal interviews. Participants were asked whether a doctor or other health professional had ever told them that they had congestive heart failure, coronary heart disease, angina, myocardial infarction, or stroke. Participants who answered “yes” to any of these questions were considered to have CVD. In the real-world cohort, the diagnosis of CVD was jointly determined by two or more specialist physicians based on blood test results and imaging findings according to ICD-10 codes, as shown in Supplementary Table S1.

### 2.4 Covariates

The baseline variables included in both the NHANES and real-world datasets were age, sex, current smoking status, current drinking status, family history of CVD, diabetes, hypertension, chronic kidney disease (CKD), chronic liver disease (CLD), lipid-lowering therapy, glucose-lowering therapy, heart rate (HR), systolic blood pressure (SBP), diastolic blood pressure (DBP), body mass index (BMI), and blood biochemical parameters, including alanine aminotransferase (ALT), aspartate aminotransferase (AST), hemoglobin (HB), total cholesterol (TC), serum creatinine (Cr), and blood urea nitrogen (BUN). Because NHANES includes participants from diverse racial and ethnic backgrounds, whereas the real-world dataset consisted exclusively of Asian participants, race/ethnicity was additionally included as a covariate in the analyses of the NHANES dataset. In addition, AD severity, as assessed by qualified physicians, was included as an additional covariate in the analyses of the real-world dataset. For models including TyG and CTI as exposure variables, BMI was included as an adjustment covariate. However, BMI was not included as an adjustment covariate in models incorporating the BMI-derived indices (TyG-BMI and CTI-BMI) to avoid potential overadjustment.

AD severity was assessed using the Eczema Area and Severity Index (EASI), a validated clinician-reported instrument that evaluates the extent and severity of eczema based on erythema, infiltration/papulation, excoriation, and lichenification across four anatomical regions. The total EASI score ranges from 0 to 72, with higher scores indicating greater disease severity. In the real-world cohort, AD severity was categorized according to established clinical criteria: mild AD was defined as EASI ≤7, moderate AD as EASI >7 to ≤21, and severe AD as EASI >21.

To evaluate potential multicollinearity among covariates, variance inflation factor (VIF) analyses were performed before multivariable regression modeling. Variables with evidence of substantial collinearity were carefully evaluated, and the final covariate sets were determined based on both clinical relevance and collinearity assessment. A VIF value <5 was considered indicative of acceptable collinearity (Supplementary Tables S2, S3).

### 2.5 Statistical analysis

According to the NHANES analytic guidelines, the complex sampling design and sampling weights were taken into account in our analyses (23). Sampling weights were calculated using the following formula: 6-year fasting subsample mobile examination center (MEC) weight = 2-year fasting subsample MEC weight/3. In this study, the distributions of variables were summarized. Continuous variables are presented as the mean with standard deviation (SD), whereas categorical variables are presented as numbers and percentages (%). Participants were divided into two groups according to the presence or absence of CVD, and the distributions of variables were compared between the two groups. Normally distributed continuous variables were compared using the independent-samples *t*-test, whereas non-normally distributed continuous variables were compared using the Wilcoxon rank-sum test. Categorical variables were compared using the chi-square test.

In this study, the TyG, TyG-BMI, CTI, and CTI-BMI indices were analyzed as both continuous and categorical variables. To further enhance the comparability among these indices, they were standardized when analyzed as continuous variables, with a mean of 0 and a standard deviation of 1. When analyzed as categorical variables, patients were divided into three groups in ascending order according to the tertiles of each index: Q1, Q2, and Q3. Three models were constructed to investigate the associations. Model 1 included no covariates. Model 2 was adjusted only for age, sex, smoking history, and drinking history. Model 3 included all covariates. Univariate and multivariable logistic regression analyses were used to calculate the odds ratios (ORs) and 95% confidence intervals (95% CIs) for different index groups.

To further validate the continuous associations of the TyG, TyG-BMI, CTI, and CTI-BMI indices with the risk of CVD, restricted cubic spline (RCS) analysis with four knots was performed to model the potential nonlinear relationships between these indices and CVD. The RCS method enables flexible modeling of associations by fitting a series of piecewise cubic polynomials, with constraints imposed at the knots to ensure smoothness at the boundaries. This approach allows the identification of potential nonlinear effects while avoiding overfitting. Subsequently, subgroup and interaction analyses were conducted to further explore potential interactions and influencing factors.

To verify the robustness of our findings and minimize the potential influence of other factors, we conducted a series of sensitivity analyses. First, observations falling within the upper and lower 5% of the distributions of the aforementioned indices were excluded prior to analysis to further reduce the influence of extreme values. Second, the analyses were repeated after missing clinical covariates were imputed using multiple imputation. Third, to account for the potential influence of AD severity, AD severity categories were additionally included as covariates in the models using the real-world dataset. Fourth, to minimize the potential confounding effects of lipid-lowering and glucose-lowering therapies, treatment status for these medications was additionally adjusted for in models based on both the real-world and NHANES datasets. Fifth, to evaluate potential overadjustment effects, fully adjusted models incorporating diabetes, hypertension, and lipid-related variables were conducted. Sixth, considering that BMI is an integral component of the BMI-derived indices (TyG-BMI and CTI-BMI), additional sensitivity analyses were performed by removing BMI adjustment from the models evaluating TyG and CTI to assess whether the associations remained robust without BMI adjustment. Finally, because atrial fibrillation (AF) and cardiovascular death were additionally included in the definition of CVD outcomes in the real-world dataset, we repeated the analyses after excluding participants with these conditions to assess whether their inclusion materially affected the results.

### 2.6 Machine learning models

To evaluate model generalizability and discriminative performance, external validation was performed between the NHANES and real-world datasets. Models were trained using the NHANES dataset and externally validated in the real-world dataset. Baseline models were constructed using age, gender, HR, family history of CVD, CKD, hypertension, CLD, current smoking status, current drinking status, ALT, AST, BUN, Cr, TC, and HB. TyG, TyG-BMI, CTI, and CTI-BMI were subsequently added separately to the baseline model to evaluate their incremental value. Seven machine learning algorithms, including logistic regression (LR), k-nearest neighbors (KNN), support vector machine (SVM), multilayer perceptron (MLP), extreme gradient boosting (XGBoost), decision tree (DT), and random forest (RF), were applied. Five-fold cross-validation and hyperparameter tuning were performed in the training dataset. Model performance was assessed using the receiver operating characteristic curve (ROC-AUC), calibration curves, precision, recall, *F*1 score, and accuracy. DeLong's test was used to compare AUC values, and the net reclassification improvement (NRI) and integrated discrimination improvement (IDI) were used to evaluate the incremental value of the four indices.

SHAP analysis was performed to interpret feature contributions in the machine learning models (24, 25). Feature importance was visualized using SHAP summary plots.

All analyses were performed using R version 4.5.1 and Python version 3.13.5. All statistical tests were two-sided, and statistical significance was determined at a *P* value of <0.05.

## 3 Results

### 3.1 Baseline characteristics

We compared the baseline characteristics between patients with and without CVD in the real-world and NHANES datasets, respectively (Supplementary Tables S4, S5). The results showed that 145 of the 454 patients with AD in the real-world dataset had prevalent CVD, whereas 75 of the 486 patients with eczema in the NHANES dataset had prevalent CVD. After applying NHANES sampling weights, the weighted prevalence of CVD among participants with eczema was approximately 12%. In both datasets, older age and higher TyG, TyG-BMI, CTI, and CTI-BMI levels were significantly associated with the occurrence of CVD. In addition, patients with CVD in the real-world dataset had a higher prevalence of CKD and higher levels of HB and TC. In the NHANES dataset, patients with CVD had higher prevalences of diabetes, hypertension, and CLD, as well as higher BMI, BUN, and Cr levels. These findings suggest that the four IR-related indices may be associated with the occurrence of CVD in patients with AD, and that age is an important confounding factor.

### 3.2 Association analyses between the four indices and CVD in patients with AD

We analyzed the associations of the TyG, TyG-BMI, CTI, and CTI-BMI indices with prevalent CVD in the real-world and NHANES datasets, respectively (Tables 1, 2). In the real-world dataset, all four indices were positively associated with prevalent CVD when analyzed either as continuous variables or tertile-based categorical variables, and these associations remained significant after adjustment for clinical covariates. In the NHANES dataset, the results were generally consistent with those of the real-world dataset, except that TyG was not significantly associated with prevalent CVD when analyzed as a tertile-based categorical variable. This discrepancy may be related to the reduced statistical power and loss of information resulting from categorizing continuous variables into tertiles.

**Table 1:** Logistic regression analysis of the associations between TyG, TyG-BMI, CTI, CTI-BMI and CVD in patients with AD in the real-word.

Models	Per 1 SD increase	*P* value	Q2 vs. Q1	*P* value	Q3 vs. Q1	*P* value
TyG						
Model 1	1.308 (1.071, 1.605)	9.15 × 10^−3^	1.797 (1.074, 3.037)	2.66 × 10^−2^	2.218 (1.353, 3.684)	1.78 × 10^−3^
Model 2	1.320 (1.077, 1.625)	8.04 × 10^−3^	1.904 (1.128, 3.248)	1.68 × 10^−2^	2.254 (1.366, 3.773)	1.68 × 10^−3^
Model 3	1.271 (1.018, 1.594)	3.58 × 10^−2^	1.995 (1.149, 3.506)	1.49 × 10^−2^	2.126 (1.248, 3.671)	6.04 × 10^−3^
TyG-BMI						
Model 1	1.349 (1.107, 1.653)	3.31 × 10^−3^	2.430 (1.450, 4.139)	8.77 × 10^−4^	2.875 (1.724, 4.880)	6.67 × 10^−5^
Model 2	1.359 (1.113, 1.666)	2.85 × 10^−3^	2.397 (1.428, 4.087)	1.08 × 10^−3^	2.899 (1.735, 4.932)	6.25 × 10^−5^
Model 3	1.410 (1.130, 1.766)	2.49 × 10^−3^	2.374 (1.373, 4.175)	2.25 × 10^−3^	3.119 (1.788, 5.552)	7.99 × 10^−5^
CTI						
Model 1	1.561 (1.268, 1.936)	3.56 × 10^−5^	2.173 (1.304, 3.671)	3.21 × 10^−3^	2.574 (1.553, 4.332)	2.94 × 10^−4^
Model 2	1.590 (1.279, 1.992)	4.00 × 10^−5^	2.182 (1.279, 3.776)	4.62 × 10^−3^	2.568 (1.513, 4.425)	5.52 × 10^−4^
Model 3	1.522 (1.204, 1.938)	5.22 × 10^−4^	2.029 (1.158, 3.604)	1.43 × 10^−2^	2.293 (1.312, 4.062)	3.93 × 10^−3^
CTI-BMI						
Model 1	1.506 (1.231, 1.855)	8.78 × 10^−5^	2.577 (1.525, 4.439)	4.95 × 10^−4^	3.406 (2.030, 5.836)	5.07 × 10^−6^
Model 2	1.517 (1.238, 1.872)	7.31 × 10^−5^	2.545 (1.504, 4.387)	6.05 × 10^−4^	3.408 (2.029, 5.849)	5.34 × 10^−6^
Model 3	1.581 (1.264, 1.992)	7.45 × 10^−5^	2.484 (1.424, 4.413)	1.56 × 10^−3^	3.520 (2.006, 6.314)	1.60 × 10^−5^

Model 1: unadjusted. Model 2: analyzed adjusted for age, gender, smoking and drinking. Model 3: analyzed adjusted for age, gender, BMI (TyG and CTI), smoking, drinking, CKD, CLD, family history of CVD, HR, HB, ALT, AST, BUN, Cr.

**Table 2:** Logistic regression analysis of the associations between TyG, TyG-BMI, CTI, CTI-BMI and CVD in patients with AD in the NHANES.

Models	Per 1 SD increase	*P* value	Q2 vs. Q1	*P* value	Q3 vs. Q1	*P* value
TyG						
Model 1	1.780 (1.355, 2.339)	1.18 × 10^−4^	1.536 (0.619, 3.814)	3.46 × 10^−1^	2.849 (1.549, 5.241)	1.27 × 10^−3^
Model 2	1.716 (1.319, 2.232)	1.89 × 10^−4^	1.183 (0.437, 3.203)	7.34 × 10^−1^	2.122 (1.164, 3.869)	1.56 × 10^−2^
Model 3	1.607 (1.107, 2.332)	1.45 × 10^−2^	1.079 (0.398, 2.928)	8.77 × 10^−1^	1.530 (0.753, 3.106)	2.29 × 10^−1^
TyG-BMI						
Model 1	1.543 (1.218, 1.954)	6.42 × 10^−4^	3.829 (1.257, 11.664)	1.94 × 10^−2^	6.256 (2.165, 18.079)	1.20 × 10^−3^
Model 2	1.598 (1.231, 2.074)	8.34 × 10^−4^	3.308 (1.018, 10.748)	4.67 × 10^−2^	5.818 (1.987, 17.039)	2.03 × 10^−3^
Model 3	1.397 (1.036, 1.885)	2.99 × 10^−2^	3.125 (0.878, 11.121)	7.65 × 10^−2^	5.131 (1.563, 16.841)	8.82 × 10^−3^
CTI						
Model 1	1.916 (1.420, 2.585)	8.15 × 10^−5^	3.237 (1.303, 8.045)	1.28 × 10^−2^	5.634 (2.599, 12.212)	5.63 × 10^−5^
Model 2	1.836 (1.360, 2.477)	2.18 × 10^−4^	2.743 (1.054, 7.138)	3.92 × 10^−2^	4.468 (2.034, 9.815)	4.66 × 10^−4^
Model 3	1.559 (1.120, 2.171)	1.04 × 10^−2^	2.467 (0.913, 6.665)	7.33 × 10^−2^	3.284 (1.351, 7.985)	1.06 × 10^−2^
CTI-BMI						
Model 1	1.523 (1.204, 1.926)	8.28 × 10^−4^	4.044 (1.255, 13.024)	2.05 × 10^−2^	6.343 (2.161, 18.618)	1.28 × 10^−3^
Model 2	1.578 (1.222, 2.039)	9.03 × 10^−4^	4.412 (1.283, 15.170)	1.99 × 10^−2^	6.459 (2.133, 19.560)	1.61 × 10^−3^
Model 3	1.371 (1.022, 1.840)	3.64 × 10^−2^	3.214 (0.872, 11.840)	7.72 × 10^−2^	4.523 (1.406, 14.550)	1.33 × 10^−2^

Model 1: unadjusted. Model 2: analyzed adjusted for age, gender, race, smoking and drinking. Model 3: analyzed adjusted for age, gender, race, BMI (TyG and CTI), smoking, drinking, CKD, CLD, family history of CVD, HR, HB, ALT, AST, BUN, Cr.

To investigate whether nonlinear associations existed between the four indices and prevalent CVD, RCS analyses were performed (Figures 1, 2). The results showed linear associations between all four indices and prevalent CVD in both the real-world and NHANES datasets, with all *P* values for nonlinearity >0.05.

**Figure 1:**
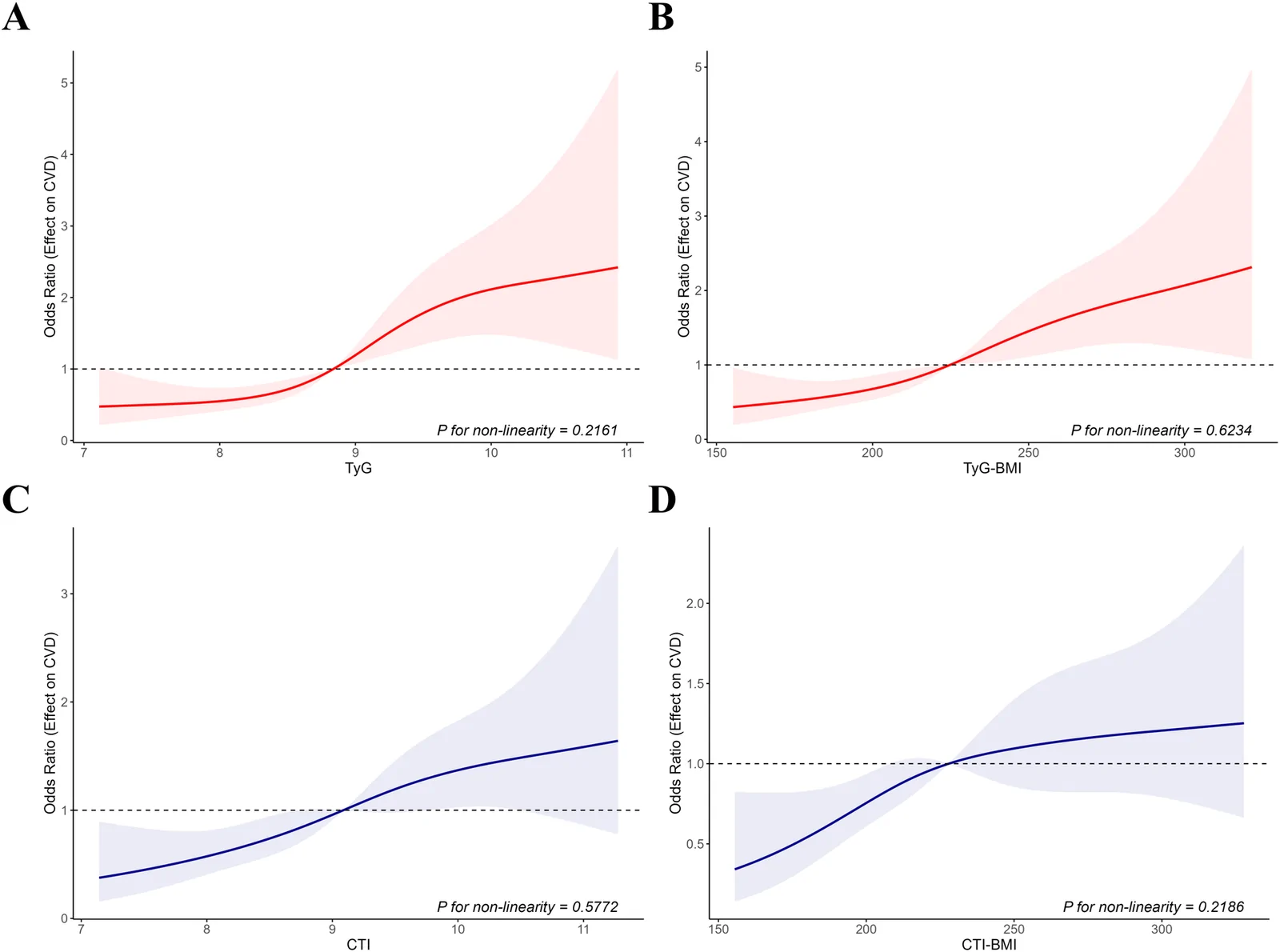
RSC analysis in the real-world. **(A)** TyG; **(B)** TyG-BMI; **(C)** CTI; **(D)** CTI-BMI.

**Figure 2:**
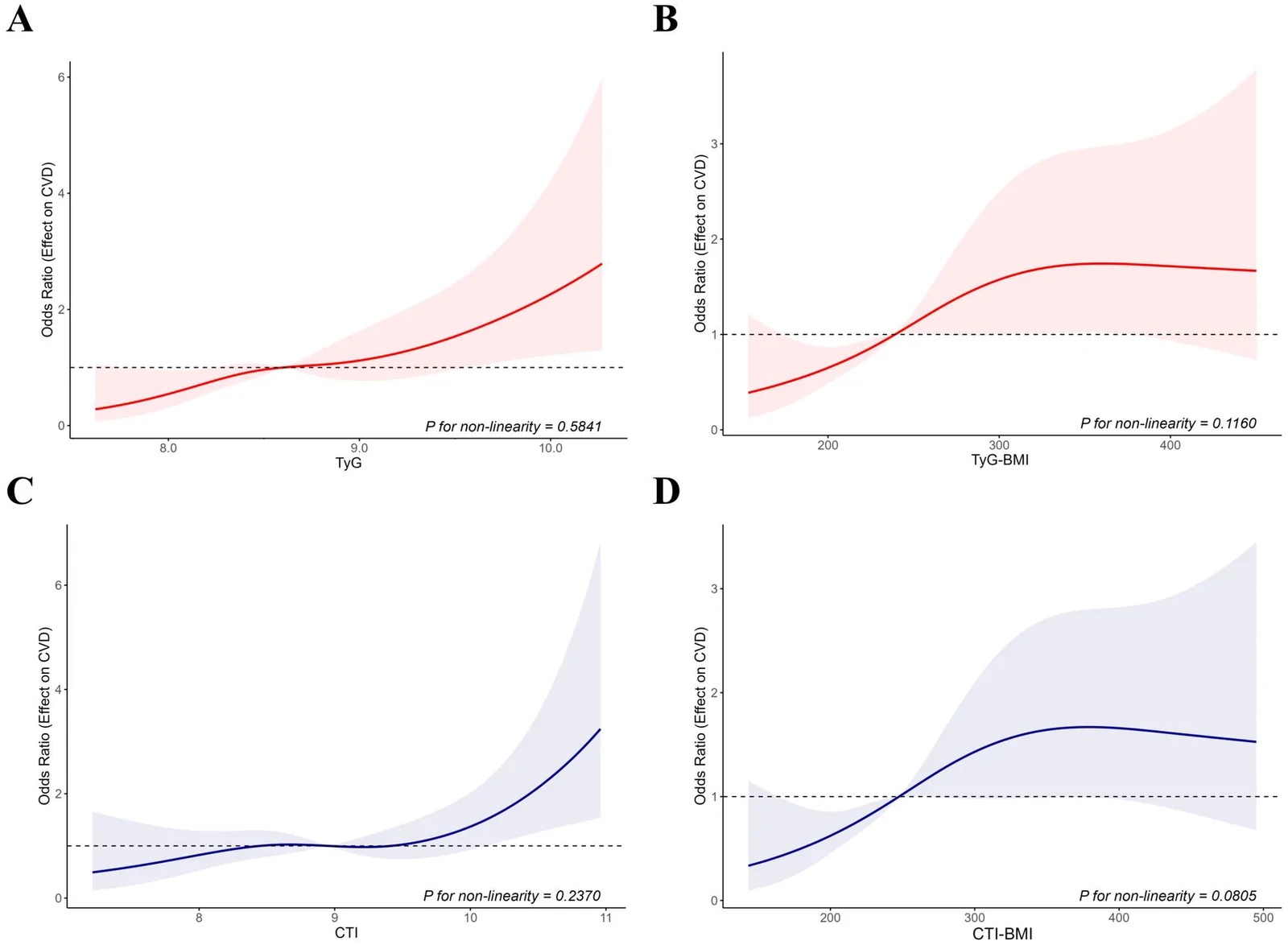
RSC analysis in the NHANES. **(A)** TyG; **(B)** TyG-BMI; **(C)** CTI; **(D)** CTI-BMI.

### 3.3 Subgroup analysis

To investigate whether the associations between the four continuous indices and CVD differed across subgroups, we performed subgroup and interaction analyses according to potential CVD risk factors. Because some subgroups in the NHANES dataset had small sample sizes, which may have led to substantial estimation errors, only selected subgroups were included in the NHANES analysis. The results from both the real-world and NHANES datasets showed that the four indices remained significantly and positively associated with CVD in most subgroups, with no significant interactions observed across subgroup factors (Supplementary Tables S6–S9). However, the associations between the four indices and CVD were attenuated in smokers, drinkers, and patients with CKD or CLD in the real-world dataset, as well as in patients with hypertension in the NHANES dataset. This attenuation may be attributable to the small sample sizes in these subgroups or to the greater contribution of these risk factors themselves to CVD compared with the four indices.

### 3.4 Sensitivity analysis

We performed several sensitivity analyses to assess the robustness of the associations between the four indices and prevalent CVD. After truncating the upper and lower 5% of extreme values, the associations between the four continuous indices and prevalent CVD remained consistent with the primary analyses in both the real-world and NHANES datasets (Supplementary Tables S10, S11). Similarly, after missing values were imputed using multiple imputation, the associations between the four indices and prevalent CVD remained largely unchanged in both datasets (Supplementary Tables S12, S13).

Furthermore, additional sensitivity analyses were performed in the real-world dataset by further adjusting for AD severity and medication use, including glucose-lowering and lipid-lowering therapies. The associations between the four indices and prevalent CVD remained robust after these additional adjustments (Supplementary Table S14). Further adjustment for diabetes, hypertension, and lipid-related variables did not materially alter the observed associations in either the real-world or NHANES datasets (Supplementary Tables S14, S15). In sensitivity analyses comparing models with and without BMI adjustment for TyG and CTI, the associations remained consistent after removing BMI adjustment, suggesting that the observed findings were not substantially influenced by potential overadjustment related to BMI (Supplementary Table S16). In addition, sensitivity analyses excluding patients with AF and cardiac death in the real-world dataset yielded consistent results, further supporting the robustness of the observed associations (Supplementary Table S17).

### 3.5 Machine learning models and SHAP analysis

The above results indicated that the four indices were significantly associated with prevalent CVD in patients with AD. To further evaluate their clinical utility and generalizability, machine learning models were developed using the NHANES dataset as the training cohort and externally validated in the real-world dataset. The discriminative performance of models incorporating each of the four indices was evaluated using ROC-AUC, calibration curves, precision, recall, *F*1 score, and accuracy.

In the external validation analysis, models incorporating CTI-BMI generally demonstrated the highest discrimination performance for prevalent CVD status. Among all evaluated models, the CTI-BMI-based model achieved the highest ROC-AUC, with the XGBoost model showing the best discrimination performance (AUC = 0.666, Baseline model AUC = 0.578, ΔAUC = 0.089, *P* value = 1.72 × 10^−3^; ΔNRI = 0.217, *P* value = 2.60 × 10^−2^; ΔIDI = 0.058, *P* value = 3.50 × 10^−2^) (Table 3). In comparison, models incorporating the other three indices showed relatively lower discrimination performance. Calibration curves and additional evaluation metrics further supported the robustness of the models (Figure 3, Table 3 and Supplementary Tables S18, S19).

**Table 3:** Comparison of model performance after adding the four IR indices to the baseline model.

Feature	Model	AUC (95% CI)	ΔAUC	*P* value	Brier score	ΔNRI (95% CI)	*P* value	ΔIDI (95% CI)	*P* value
Base	LR	0.485 (0.426–0.544)			0.261				
	KNN	0.480 (0.427–0.533)			0.277				
	SVC	0.591 (0.536–0.647)			0.237				
	MLP	0.445 (0.386–0.504)			0.314				
	XGboost	0.578 (0.521–0.634)			0.263				
	DT	0.548 (0.496–0.599)			0.438				
	RF	0.598 (0.544–0.653)			0.216				
Base + TyG	LR	0.517 (0.459–0.575)	0.032	3.92 × 10^−2^	0.263	0.170 (0.014–0.326)	3.00 × 10^−2^	0.015 (−0.004 to 0.035)	1.26 × 10^−1^
	KNN	0.493 (0.439–0.548)	0.014	4.16 × 10^−1^	0.269	0.067 (−0.022 to 0.156)	1.51 × 10^−1^	0.014 (−0.008 to 0.035)	2.33 × 10^−1^
	SVC	0.638 (0.584–0.691)	0.046	8.94 × 10^−3^	0.236	0.299 (0.167–0.430)	1.00 × 10^−3^	0.005 (0.003–0.008)	1.00 × 10^−3^
	MLP	0.616 (0.560–0.671)	0.171	4.93 × 10^−8^	0.277	0.319 (0.155–0.487)	2.00 × 10^−3^	0.098 (0.030–0.164)	4.00 × 10^−3^
	XGboost	0.633 (0.579–0.688)	0.056	1.55 × 10^−2^	0.256	0.190 (−0.009 to 0.388)	6.70 × 10^−2^	0.064 (0.018–0.114)	1.30 × 10^−2^
	DT	0.546 (0.492–0.599)	−0.002	9.54 × 10^−1^	0.375	−0.131 (−0.239 to −0.022)	2.00 × 10^−2^	−0.075 (−0.153 to 0.002)	5.60 × 10^−2^
	RF	0.604 (0.550–0.658)	0.006	6.45 × 10^−1^	0.213	0.111 (−0.082 to 0.293)	2.50 × 10^−1^	0.008 (−0.001 to 0.016)	8.50 × 10^−2^
Base + TyG-BMI	LR	0.527 (0.468–0.585)	0.041	8.73 × 10^−6^	0.252	0.284 (0.134–0.446)	1.00 × 10^−3^	0.008 (−0.006 to 0.020)	2.28 × 10^−1^
	KNN	0.519 (0.466–0.572)	0.039	9.40 × 10^−4^	0.273	0.126 (0.047–0.204)	6.00 × 10^−3^	0.026 (0.010–0.042)	5.00 × 10^−3^
	SVC	0.614 (0.558–0.669)	0.022	2.35 × 10^−2^	0.232	0.071 (−0.095 to 0.250)	4.19 × 10^−1^	0.002 (0.001–0.003)	1.00 × 10^−3^
	MLP	0.645 (0.589–0.700)	0.200	2.98 × 10^−10^	0.297	0.169 (0.016–0.313)	3.20 × 10^−2^	0.125 (0.061–0.189)	1.00 × 10^−3^
	XGboost	0.653 (0.601–0.706)	0.076	9.76 × 10^−3^	0.246	0.193 (0.008–0.367)	4.50 × 10^−2^	0.021 (−0.033 to 0.071)	4.39 × 10^−1^
	DT	0.511 (0.456–0.567)	−0.036	3.03 × 10^−1^	0.300	−0.238 (−0.352 to −0.120)	1.00 × 10^−3^	−0.117 (−0.203 to −0.033)	7.00 × 10^−3^
	RF	0.650 (0.598–0.702)	0.052	1.79 × 10^−4^	0.212	0.252 (0.061–0.444)	1.30 × 10^−2^	0.012 (0.005–0.019)	1.00 × 10^−3^
Base + CTI	LR	0.533 (0.475–0.591)	0.048	1.72 × 10^−3^	0.251	0.235 (0.039–0.418)	2.00 × 10^−2^	0.019 (0.002–0.036)	3.40 × 10^−2^
	KNN	0.478 (0.425–0.532)	−0.002	9.14 × 10^−1^	0.275	0.023 (−0.062 to 0.108)	6.33 × 10^−1^	0.003 (−0.016 to 0.022)	7.71 × 10^−1^
	SVC	0.623 (0.568–0.677)	0.031	3.22 × 10^−3^	0.234	0.436 (0.256–0.612)	1.00 × 10^−3^	0.005 (0.003–0.007)	1.00 × 10^−3^
	MLP	0.656 (0.600–0.711)	0.211	1.04 × 10^−1^	0.266	0.186 (0.071–0.297)	4.00 × 10^−3^	0.191 (0.113–0.264)	1.00 × 10^−3^
	XGboost	0.634 (0.581–0.687)	0.056	1.66 × 10^−2^	0.251	0.158 (−0.035 to 0.345)	1.13 × 10^−1^	0.035 (−0.012 to 0.083)	1.43 × 10^−1^
	DT	0.560 (0.507–0.613)	0.012	7.10 × 10^−1^	0.280	−0.101 (−0.224 to 0.026)	1.14 × 10^−1^	−0.057 (−0.129 to 0.018)	1.40 × 10^−1^
	RF	0.628 (0.575–0.682)	0.030	2.29 × 10^−2^	0.211	0.215 (0.020–0.399)	3.10 × 10^−2^	0.015 (0.006–0.025)	5.00 × 10^−3^
Base + CTI-BMI	LR	0.524 (0.466–0.583)	0.039	2.24 × 10^−5^	0.262	0.274 (0.112–0.447)	1.00 × 10^−3^	0.008 (−0.006 to 0.019)	2.25 × 10^−1^
	KNN	0.512 (0.460–0.565)	0.032	2.39 × 10^−2^	0.272	0.111 (0.025–0.197)	1.10 × 10^−2^	0.022 (0.004–0.040)	1.90 × 10^−2^
	SVC	0.616 (0.560–0.671)	0.024	2.12 × 10^−2^	0.236	−0.030 (−0.211 to 0.162)	7.39 × 10^−1^	0.002 (0.001–0.003)	1.00 × 10^−3^
	MLP	0.650 (0.595–0.705)	0.205	1.28 × 10^−10^	0.276	0.112 (−0.008 to 0.233)	6.60 × 10^−2^	0.165 (0.093–0.237)	1.00 × 10^−3^
	XGboost	0.666 (0.614–0.719)	0.089	1.72 × 10^−3^	0.241	0.217 (0.031–0.401)	2.60 × 10^−2^	0.058 (0.004–0.110)	3.50 × 10^−2^
	DT	0.585 (0.531–0.639)	0.038	2.90 × 10^−1^	0.289	−0.154 (−0.265 to −0.043)	1.10 × 10^−2^	−0.043 (−0.121 to 0.033)	2.84 × 10^−1^
	RF	0.657 (0.604–0.709)	0.058	2.93 × 10^−4^	0.211	0.272 (0.068–0.464)	7.00 × 10^−3^	0.016 (0.007–0.024)	1.00 × 10^−3^

**Figure 3:**
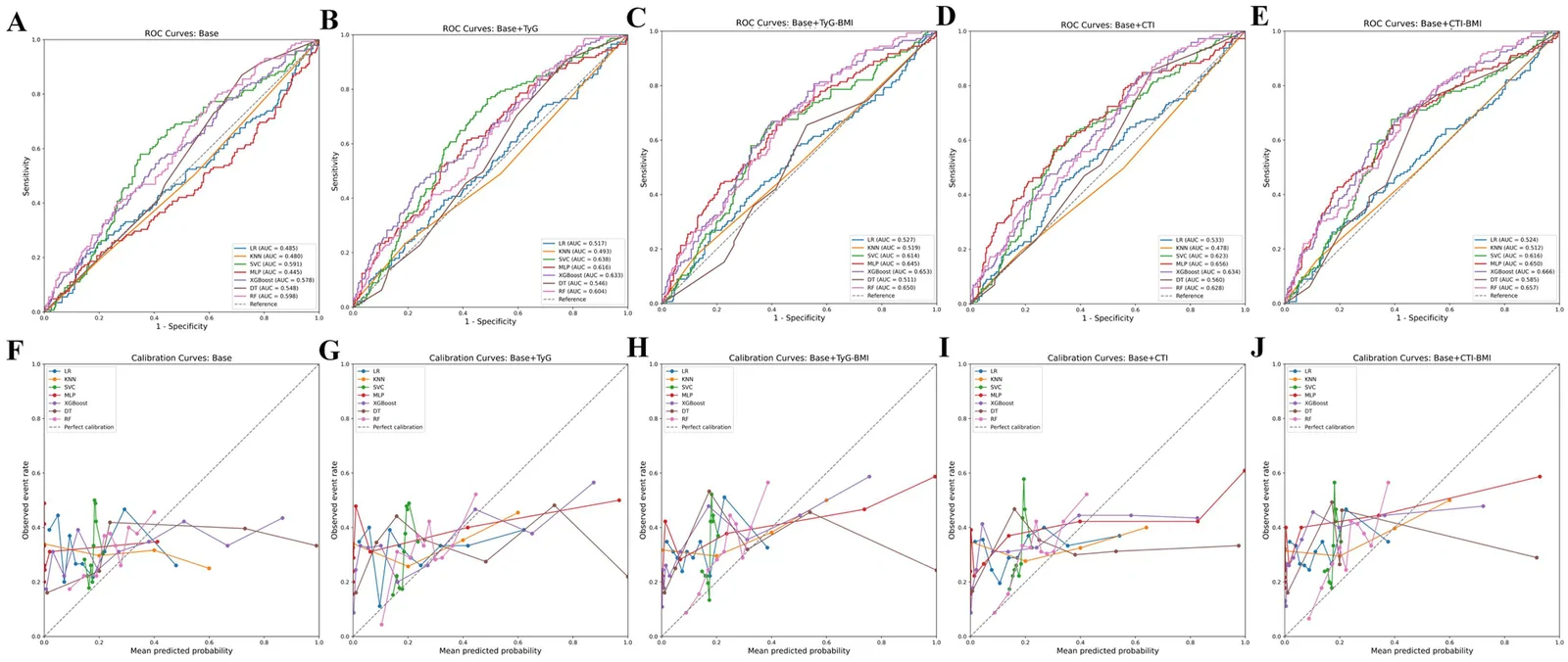
ROC curves and calibration curves of machine learning models. **(A,F)** Base; **(B,G)** Base + TyG; **(C,H)** Base + TyG-BMI; **(D,I)** Base + CTI; **(E,J)** Base + CTI-BMI.

SHAP analysis further demonstrated the relative contribution of the four indices to model discrimination, with CTI-BMI showing the greatest contribution among the evaluated indices. SHAP analysis further showed that CTI-BMI had the highest contribution among the four indices (Supplementary Figures S1), supporting the above findings.

## 4 Discussion

Increasing evidence indicates that, beyond local cutaneous manifestations, patients with AD exhibit systemic inflammatory abnormalities that may be associated with increased cardiovascular burden. Therefore, identifying biomarkers associated with prevalent CVD among patients with AD may provide additional insights into cardiovascular burden assessment. In this study, we found that the TyG, TyG-BMI, CTI, and CTI-BMI indices were positively and linearly associated with prevalent CVD status in patients with AD. These associations were consistent across most subgroups and remained robust in sensitivity analyses. We further developed machine learning models incorporating these indices to evaluate their discriminative performance for prevalent CVD status. Among the evaluated indices, CTI-BMI demonstrated the highest discriminative ability, although the overall incremental discrimination remained modest. Our findings suggest that these composite indices may serve as potential adjunctive markers for assessing cardiovascular burden among patients with AD.

Existing studies have suggested an association between AD and cardiovascular burden; however, the relationship remains complex and incompletely understood. A large cohort study including approximately 40,000 patients with AD and 40,000 individuals without AD reported that adults with newly diagnosed AD had a higher risk of subsequent CVD events (5). An NHANES-based analysis also demonstrated an association between AD and myocardial infarction (MI) (26). In addition, a meta-analysis showed that greater AD severity was associated with a higher prevalence of cardiovascular comorbidities, including MI, angina, and cardiovascular death (10). These findings suggest that chronic inflammatory burden associated with AD may contribute to cardiovascular vulnerability.

Nevertheless, the relationship between AD and CVD remains controversial. A Mendelian randomization study using data from European populations found no significant evidence supporting a causal effect of AD on CVD outcomes, suggesting that shared genetic susceptibility alone may not fully explain the epidemiological associations observed in previous studies (27). Similarly, a Danish nationwide cohort study reported that the association between AD and CVD was substantially attenuated and became non-significant after adjustment for traditional cardiovascular risk factors and comorbidities, indicating that conventional risk factors may partially account for the observed association (28). These inconsistent findings may be attributable to differences in population characteristics, ethnicity, disease severity, treatment exposure, study design, and covariate adjustment strategies.

Beyond the direct associations between AD and CVD, increasing evidence suggests that AD may be associated with increased cardiovascular vulnerability through systemic inflammatory processes and related metabolic alterations. Although AD has traditionally been considered a skin-limited inflammatory disorder, moderate-to-severe AD is increasingly recognized as a systemic inflammatory condition characterized by persistent immune activation and circulating inflammatory abnormalities. Chronic activation of inflammatory pathways may promote endothelial dysfunction, oxidative stress, and vascular inflammation, thereby contributing to a pro-atherogenic environment. Previous studies have demonstrated that patients with AD, particularly those with more severe disease, exhibit increased cardiovascular burden and subclinical atherosclerotic changes, suggesting that systemic inflammatory activity may represent an important link between cutaneous inflammation and cardiovascular impairment (29).

Several potential pathological mechanisms may underlie the observed association between AD and CVD. Recent evidence suggests that Th2-mediated cytokines, such as IL-4 and IL-13, which play central roles in AD pathogenesis, may be involved in vascular inflammation and atherosclerotic processes by promoting endothelial dysfunction and immune cell infiltration into the arterial wall (30). In addition, AD-related systemic inflammation may be associated with alterations in platelet activity, coagulation balance, and fibrinolytic function, which could contribute to an unfavorable cardiovascular environment (31). Furthermore, epithelial cell-derived alarmin thymic stromal lymphopoietin (TSLP) has been detected in both coronary artery disease (CAD) and AD and may participate in immune regulation across multiple organs (32). Collectively, these findings provide biological plausibility that systemic inflammatory pathways associated with AD may partly explain the increased cardiovascular burden observed in some patients with AD. However, whether these inflammatory pathways directly mediate CVD development remains uncertain, particularly given the potential influence of traditional cardiovascular risk factors and treatment-related factors. In addition to immune-mediated vascular effects, chronic inflammatory activation in AD may also influence metabolic homeostasis and insulin signaling, representing another potential link between AD and cardiovascular burden.

The TyG, TyG-BMI, CTI, and CTI-BMI indices incorporate parameters related to glucose metabolism, lipid metabolism, inflammation, and adiposity, and have been investigated as surrogate indicators associated with insulin resistance-related metabolic abnormalities. However, whether these indices accurately reflect insulin resistance specifically in patients with AD remains insufficiently established. Insulin resistance is a core component of metabolic syndrome (MetS), which represents a cluster of metabolic abnormalities and is associated with an increased prevalence of diabetes, atherosclerotic cardiovascular disease, and other cardiovascular complications (33). Several studies have suggested an association between AD and MetS, potentially due to shared inflammatory mechanisms and metabolic dysregulation (34).

Previous studies in patients with psoriasis have demonstrated that TyG and TyG-BMI were associated with cardiovascular burden (35). Given the shared features of chronic systemic inflammation between psoriasis and AD, these indices may also capture metabolic abnormalities relevant to cardiovascular involvement in patients with AD. In addition to TyG-related indices, CTI incorporates CRP as an inflammatory component and has been investigated in relation to cardiovascular outcomes (36). Therefore, these composite indices may provide additional information regarding the interplay between metabolic disturbances, systemic inflammation, and cardiovascular burden beyond traditional risk factors. However, given the cross-sectional design of the present study, these indices should be interpreted as markers associated with prevalent CVD status rather than validated predictors of future cardiovascular events.

This study analyzed real-world data from the First Affiliated Hospital of Harbin Medical University and NHANES data to investigate the associations between four metabolic-inflammatory indices and prevalent CVD status among patients with AD. The consistency of the findings across two independent datasets from different sources provided supportive evidence for the robustness of the observed associations. In addition, RCS analyses, subgroup analyses, and sensitivity analyses further supported the stability of the results. Furthermore, machine learning models incorporating these indices were developed and externally validated to evaluate their discriminative performance for prevalent CVD status. Our findings suggest that these readily available and cost-effective indices may provide complementary information for cardiovascular burden assessment in patients with AD; however, prospective longitudinal studies are needed to determine whether these indices can predict future cardiovascular events.

This study has several limitations. First, because both datasets were cross-sectional, temporal relationships between the four indices and prevalent CVD could not be established. In the real-world cohort, blood samples were collected after CVD diagnosis, as CVD was recorded as a pre-existing comorbidity during AD-related clinical visits rather than the primary reason for enrollment. Similarly, in the NHANES dataset, CVD status was based on self-reported physician-diagnosed cardiovascular conditions, indicating that laboratory measurements were obtained after CVD diagnosis. Therefore, inflammatory and metabolic parameters, particularly CRP-based indices, may have been influenced by existing cardiovascular disease or related treatments, and reverse causality cannot be completely excluded. Prospective longitudinal studies with repeated measurements before disease onset are required to determine whether these indices can predict future cardiovascular events. Second, although we performed multiple sensitivity analyses, including additional adjustment for AD severity and medication-related factors in the real-world cohort, residual confounding could not be completely excluded. In particular, detailed information regarding AD duration, cumulative exposure, and dose intensity of systemic therapies was unavailable and could not be fully accounted for. This limitation is particularly relevant because previous studies have demonstrated that cumulative oral corticosteroid exposure in patients with moderate-to-severe AD is independently associated with insulin resistance and metabolic syndrome (37). Therefore, the potential influence of long-term systemic treatment exposure on metabolic and inflammatory profiles cannot be completely excluded in our study. Future prospective studies incorporating detailed treatment history and cumulative medication exposure are warranted to further clarify these relationships. Third, although TyG, TyG-BMI, CTI, and CTI-BMI have been widely investigated as indices associated with insulin resistance-related metabolic abnormalities, direct measurements of insulin resistance, such as HOMA-IR, were unavailable in our datasets. Therefore, these indices should be interpreted as composite indicators incorporating metabolic, inflammatory, and adiposity-related information rather than validated direct measures of insulin resistance in patients with AD. Fourth, although reciprocal validation between the NHANES and real-world datasets improved the robustness and generalizability of our findings, the number of patients with AD and prevalent CVD remained relatively limited, particularly in subgroup analyses and machine learning validation. Larger prospective studies are warranted to further validate the clinical utility of these indices. Fifth, AD status in the NHANES dataset was based on self-reported physician-diagnosed eczema rather than dermatologist-confirmed AD, which may have resulted in potential misclassification and should be considered when interpreting the findings. Sixth, in the real-world cohort, blood samples were collected after the diagnosis of CVD, as CVD status was obtained from patients' previous medical history during AD-related clinical visits. Patients in this cohort primarily sought medical care for AD, whereas CVD was considered a pre-existing comorbidity rather than the primary reason for enrollment. Therefore, the laboratory measurements, including inflammatory and metabolic parameters, were obtained after CVD diagnosis. Similarly, in the NHANES dataset, CVD status was based on self-reported physician-diagnosed cardiovascular conditions, indicating that blood samples were also collected after the occurrence or diagnosis of CVD. This temporal sequence should be considered when interpreting the associations between CRP-based indices and prevalent CVD, as reverse causality cannot be completely excluded. Finally, the relatively high proportion of patients with CVD in the real-world dataset may have introduced potential selection bias and limited the generalizability of our findings. The real-world cohort was recruited from a tertiary hospital in Harbin, Heilongjiang Province, China, rather than from a population-based community survey. Previous nationwide population-based evidence has indicated that Northeast China has a relatively higher cardiovascular risk profile compared with several other regions of China, with the proportion of individuals at high cardiovascular disease risk among adults aged 35–75 years reported to be 12.6% in this region (38). This regional background may partially explain the relatively higher cardiovascular burden observed in our study population; however, this estimate is not directly comparable with the CVD prevalence observed in our hospital-based cohort due to differences in study design, outcome definitions, and population characteristics. In addition, because the real-world data were obtained from individuals who voluntarily sought medical care rather than through population-based screening, patients with cardiovascular conditions or greater disease burden may have been more likely to seek medical attention and undergo clinical evaluation. Therefore, the observed higher prevalence of CVD in the real-world cohort may partly reflect referral patterns, healthcare-seeking behavior, and spectrum bias. Although these potential biases do not invalidate the observed associations, caution is warranted when interpreting the generalizability of our findings to the broader population.

## 5 Conclusion

This study demonstrated that the TyG, TyG-BMI, CTI, and CTI-BMI indices were significantly associated with prevalent CVD status among patients with AD and showed modest discriminative performance in machine learning models. These indices are simple, cost-effective, and readily available in clinical settings, suggesting their potential value as adjunctive markers for cardiovascular burden assessment in patients with AD. Future multicenter prospective studies are warranted to determine whether these indices can provide predictive value for future cardiovascular events and to further clarify their clinical relevance in cardiovascular risk assessment and individualized management among patients with AD.

## Data Availability

Publicly available datasets were analyzed in this study. This data can be found here: https://wwwn.cdc.gov/nchs/nhanes/default.aspx.
