# Supplementary material for: Association between insulin resistance-related indices and cardiovascular disease in patients with atopic dermatitis: mutual validation using real-world and NHANES data
Source: Front Cardiovasc Med. 2026 Sep 29;13:1903179. doi: 10.3389/fcvm.2026.1903179 (PMC13658099; doi:10.3389/fcvm.2026.1903179)
Supplement: Supplementary file 1 [file Datasheet1.docx]

| **Figure S1 SHAP analysis results**    **Table S1 Cardiovascular diseases and corresponding ICD-10 codes** | |
| --- | --- |
| **Variable** | **Definition (ICD-10)** |
| **Stroke** | Ischemic stroke: I63; -Lacunar cerebral infarction: I63.801;  -Non-lacunar cerebral infarction: Types of cerebral infarction other than lacunar infarction. Intracerebral hemorrhage: I61, I62; Subarachnoid hemorrhage: I60;  Unspecified stroke: I64; |
| **Ischemic heart disease** | Ischemic heart disease  Angina pectoris: I20; Acute myocardial infarction: I21; Subsequent myocardial infarction: I22; Certain current complications following acute  myocardial infarction: I23; Other acute ischemic heart diseases: I24; Chronic ischemic heart disease: I25; |
| **Heart failure** | Hypertensive heart disease with (congestive) heart failure: I11.0; Hypertensive heart and kidney disease accompanied by (congestive) heart failure: I13.0; Hypertensive heart and kidney disease are accompanied by (congestive) heart failure and  14 kidney failure: I13.2; Congestive Heart Failure: I50; Left ventricular heart-failure: I50.1; Unspecified heart failure: I50.9; |
| **Atrial fibrillation** | I48; |

| **Table S2 Covariate selection for inclusion in the real-world dataset** | |
| --- | --- |
| **Covariate** | **VIF** |
| **Age** | 1.224 |
| **Sex** | 1.469 |
| **AD severity** | 1.166 |
| **Smoking** | 1.477 |
| **Drinking** | 1.488 |
| **Hypertension** | 1.134 |
| **Diabetes** | 5.878 |
| **CKD** | 1.390 |
| **CLD** | 1.210 |
| **Glucose-lowering** | 5.801 |
| **Lipid-lowering** | 1.103 |
| **Family history of CVD** | 1.084 |
| **HR** | 1.207 |
| **SBP** | 1.656 |
| **DBP** | 2.102 |
| **BMI** | 1.236 |
| **ALT** | 2.086 |
| **AST** | 2.147 |
| **BUN** | 2.568 |
| **Cr** | 2.654 |
| **TC** | 1.223 |
| **HB** | 1.577 |
| **CRP** | 1.108 |
|  | |

| **Table S3 Covariate selection for inclusion in the NHANES** | |
| --- | --- |
| **Covariate** | **VIF** |
| **Age** | 1.397 |
| **Sex** | 1.825 |
| **Race** | 1.191 |
| **Smoking** | 1.209 |
| **Drinking** | 1.236 |
| **Hypertension** | 1.418 |
| **Diabetes** | 4.534 |
| **CKD** | 1.628 |
| **CLD** | 1.221 |
| **dt** | 4.560 |
| **dc** | 1.369 |
| **CVDJ** | 1.094 |
| **HR** | 1.308 |
| **SBP** | 1.423 |
| **DBP** | 1.266 |
| **BMI** | 1.485 |
| **ALT** | 3.014 |
| **AST** | 3.023 |
| **BUN** | 1.393 |
| **Cr** | 1.898 |
| **TC** | 1.110 |
| **HB** | 1.783 |
| **CRP** | 1.303 |
|  | |

| **Table S4 General characteristics of participants in the real-world** | | | | |
| --- | --- | --- | --- | --- |
| **Characteristic** | **Non-CVD (N = 309)** | **CVD (N = 145)** | **Total (N = 454)** | **P value** |
| **Sex** |  |  |  |  |
| Female | 106 (34.3%) | 59 (40.7%) | 165 (36.3%) | 0.187 |
| Male | 203 (65.7%) | 86 (59.3%) | 289 (63.7%) |  |
| **Age** | 42.398 (6.890) | 46.772 (6.898) | 43.795 (7.181) | < 0.001 |
| **AD severity** |  |  |  |  |
| Mild | 186 (60.2%) | 95 (65.5%) | 281 (61.9%) | 0.51 |
| Moderate | 97 (31.4%) | 38 (26.2%) | 135 (29.7%) |  |
| Severe | 26 (8.4%) | 12 (8.3%) | 38 (8.4%) |  |
| **Smoking** |  |  |  |  |
| No | 250 (80.9%) | 119 (82.1%) | 369 (81.3%) | 0.767 |
| Yes | 59 (19.1%) | 26 (17.9%) | 85 (18.7%) |  |
| **Drinking** |  |  |  |  |
| No | 256 (82.8%) | 125 (86.2%) | 381 (83.9%) | 0.364 |
| Yes | 53 (17.2%) | 20 (13.8%) | 73 (16.1%) |  |
| **Hypertension** |  |  |  |  |
| No | 216 (69.9%) | 93 (64.1%) | 309 (68.1%) | 0.219 |
| Yes | 93 (30.1%) | 52 (35.9%) | 145 (31.9%) |  |
| **Diabetes** |  |  |  |  |
| No | 201 (65.0%) | 82 (56.6%) | 283 (62.3%) | 0.082 |
| Yes | 108 (35.0%) | 63 (43.4%) | 171 (37.7%) |  |
| **CKD** |  |  |  |  |
| No | 255 (82.5%) | 83 (57.2%) | 338 (74.4%) | < 0.001 |
| Yes | 54 (17.5%) | 62 (42.8%) | 116 (25.6%) |  |
| **CLD** |  |  |  |  |
| No | 267 (86.4%) | 114 (78.6%) | 381 (83.9%) | 0.035 |
| Yes | 42 (13.6%) | 31 (21.4%) | 73 (16.1%) |  |
| **Glucose-lowering** |  |  |  |  |
| No | 205 (66.3%) | 97 (66.9%) | 302 (66.5%) | 0.907 |
| Yes | 104 (33.7%) | 48 (33.1%) | 152 (33.5%) |  |
| **Lipid-lowering** |  |  |  |  |
| No | 210 (68.0%) | 91 (62.8%) | 301 (66.3%) | 0.274 |
| Yes | 99 (32.0%) | 54 (37.2%) | 153 (33.7%) |  |
| **Family history of CVD** |  |  |  |  |
| No | 271 (87.7%) | 123 (84.8%) | 394 (86.8%) | 0.399 |
| Yes | 38 (12.3%) | 22 (15.2%) | 60 (13.2%) |  |
| **HR** | 86.592 (16.196) | 84.600 (16.426) | 85.956 (16.278) | 0.224 |
| **SBP (mmHg)** | 133.764 (20.919) | 137.021 (24.156) | 134.804 (22.030) | 0.142 |
| **DBP (mmHg)** | 76.362 (14.186) | 72.552 (15.577) | 75.145 (14.735) | 0.01 |
| **BMI (kg/m2)** | 24.190 (3.799) | 25.891 (3.564) | 24.733 (3.805) | < 0.001 |
| **ALT (IU/L)** | 28.133 (46.101) | 31.132 (72.071) | 29.091 (55.660) | 0.593 |
| **AST (IU/L)** | 27.110 (50.316) | 35.174 (68.806) | 29.685 (56.925) | 0.16 |
| **BUN (mmol/L)** | 7.340 (4.057) | 8.109 (4.393) | 7.585 (4.178) | 0.067 |
| **Cr (mg/dL)** | 85.031 (75.937) | 100.170 (92.657) | 89.866 (81.851) | 0.066 |
| **HB (g/L)** | 133.532 (24.215) | 137.969 (20.515) | 136.552 (21.837) | 0.043 |
| **TC (mmol/L)** | 4.384 (1.535) | 4.801 (1.385) | 4.668 (1.446) | 0.004 |
| **CRP (mg/dL)** | 2.917 (3.457) | 5.537 (7.073) | 3.754 (5.052) | < 0.001 |
| **GLU (mg/dL)** | 128.484 (66.591) | 134.369 (66.891) | 130.364 (66.670) | 0.381 |
| **TG (mg/dL)** | 136.901 (124.495) | 153.742 (136.454) | 142.279 (128.525) | 0.193 |
| **TyG** | 8.699 (0.904) | 8.928 (0.761) | 8.772 (0.866) | 0.008 |
| **TyG-BMI** | 219.244 (40.731) | 231.329 (38.466) | 223.104 (40.375) | 0.003 |
| **CTI** | 8.959 (1.012) | 9.383 (0.899) | 9.094 (0.996) | < 0.001 |
| **CTI-BMI** | 225.717 (42.608) | 243.075 (41.645) | 231.261 (43.026) | < 0.001 |
| Continuous variables are presented as mean (SD), and categorical variables as n (%). | | | | |

| **Table S5 General characteristics of participants in the NAHNES** | | | | |
| --- | --- | --- | --- | --- |
| **Characteristic** | **Non-CVD (N = 411)** | **CVD (N = 75)** | **Total (N = 486)** | **P value** |
| **Sex** |  |  |  |  |
| Female | 245 (59.6%) | 43 (57.3%) | 288 (59.3%) | 0.712 |
| Male | 166 (40.4%) | 32 (42.7%) | 198 (40.7%) |  |
| **Age** | 38.292 (11.242) | 47.133 (7.847) | 39.656 (11.245) | < 0.001 |
| **Race** |  |  |  |  |
| Mexican American | 52 (12.7%) | 8 (10.7%) | 60 (12.3%) | 0.514 |
| Other Hispanic | 14 (3.4%) | 3 (4.0%) | 17 (3.5%) |  |
| Non-Hispanic White | 261 (63.5%) | 44 (58.7%) | 305 (62.8%) |  |
| Non-Hispanic Black | 66 (16.1%) | 18 (24.0%) | 84 (17.3%) |  |
| Other Race | 18 (4.4%) | 2 (2.7%) | 20 (4.1%) |  |
| **Smoking** |  |  |  |  |
| No | 293 (71.3%) | 51 (68.0%) | 344 (70.8%) | 0.565 |
| Yes | 118 (28.7%) | 24 (32.0%) | 142 (29.2%) |  |
| **Drinking** |  |  |  |  |
| No | 122 (29.7%) | 30 (40.0%) | 152 (31.3%) | 0.076 |
| Yes | 289 (70.3%) | 45 (60.0%) | 334 (68.7%) |  |
| **Hypertension** |  |  |  |  |
| No | 337 (82.0%) | 40 (53.3%) | 377 (77.6%) | < 0.001 |
| Yes | 74 (18.0%) | 35 (46.7%) | 109 (22.4%) |  |
| **Diabetes** |  |  |  |  |
| No | 394 (95.9%) | 62 (82.7%) | 456 (93.8%) | < 0.001 |
| Yes | 17 (4.1%) | 13 (17.3%) | 30 (6.2%) |  |
| **CKD** |  |  |  |  |
| No | 405 (98.5%) | 74 (98.7%) | 479 (98.6%) | 0.933 |
| Yes | 6 (1.5%) | 1 (1.3%) | 7 (1.4%) |  |
| **CLD** |  |  |  |  |
| No | 389 (94.6%) | 65 (86.7%) | 454 (93.4%) | 0.01 |
| Yes | 22 (5.4%) | 10 (13.3%) | 32 (6.6%) |  |
| **Glucose-lowering** |  |  |  |  |
| No | 13 (3.2%) | 10 (13.3%) | 23 (4.7%) | < 0.001 |
| Yes | 398 (96.8%) | 65 (86.7%) | 463 (95.3%) |  |
| **Lipid-lowering** |  |  |  |  |
| No | 27 (6.6%) | 30 (40.0%) | 57 (11.7%) | < 0.001 |
| Yes | 384 (93.4%) | 45 (60.0%) | 429 (88.3%) |  |
| **Family history of CVD** |  |  |  |  |
| No | 55 (13.4%) | 25 (33.3%) | 80 (16.5%) | < 0.001 |
| Yes | 356 (86.6%) | 50 (66.7%) | 406 (83.5%) |  |
| **HR** | 73.567 (12.597) | 73.600 (13.295) | 73.572 (12.693) | 0.983 |
| **SBP (mmHg)** | 116.769 (14.788) | 121.533 (16.376) | 117.504 (15.124) | 0.012 |
| **DBP (mmHg)** | 71.737 (11.827) | 74.480 (13.605) | 72.160 (12.144) | 0.072 |
| **BMI (kg/m2)** | 28.568 (7.279) | 32.000 (7.073) | 29.097 (7.346) | < 0.001 |
| **ALT (IU/L)** | 25.129 (16.629) | 27.707 (14.822) | 25.527 (16.375) | 0.21 |
| **AST (IU/L)** | 24.214 (12.141) | 25.507 (9.867) | 24.414 (11.819) | 0.384 |
| **BUN (mmol/L)** | 4.091 (1.477) | 4.575 (1.697) | 4.165 (1.521) | 0.011 |
| **Cr (mg/dL)** | 73.946 (17.611) | 85.571 (93.837) | 75.740 (40.291) | 0.021 |
| **HB (g/L)** | 142.922 (16.134) | 142.973 (13.046) | 142.930 (15.685) | 0.979 |
| **TC (mmol/L)** | 198.214 (42.373) | 204.693 (43.569) | 199.214 (42.578) | 0.226 |
| **CRP (mg/dL)** | 5.078 (9.601) | 7.200 (11.020) | 5.406 (9.851) | 0.086 |
| **GLU (mg/dL)** | 97.095 (24.871) | 112.129 (43.794) | 99.415 (29.071) | < 0.001 |
| **TG (mg/dL)** | 137.061 (117.344) | 172.493 (145.819) | 142.529 (122.674) | 0.021 |
| **TyG** | 8.611 (0.599) | 8.945 (0.686) | 8.662 (0.624) | < 0.001 |
| **TyG-BMI** | 247.154 (70.054) | 287.286 (73.858) | 253.347 (72.053) | < 0.001 |
| **CTI** | 8.917 (0.897) | 9.441 (0.959) | 8.998 (0.926) | < 0.001 |
| **CTI-BMI** | 257.950 (83.460) | 304.496 (86.510) | 265.133 (85.520) | < 0.001 |
| Continuous variables are presented as mean (SD), and categorical variables as n (%). | | | | |

| **Table S6 Subgroup analysis of the associations of TyG and TyG-BMI with CVD in patients with AD in the real-world** | | | | | | | |
| --- | --- | --- | --- | --- | --- | --- | --- |
| **Classification** |  | **TyG** | | | **TyG-BMI** | | |
|  | **N** | **OR (95%CI)** | **P.value** | **Interaction P** | **OR (95%CI)** | **P.value** | **Interaction P** |
| **Age** |  |  |  |  |  |  |  |
| ≥45 | 224 | 1.426 (1.129 , 1.815 ) | 3.34E-03 | 9.76E-01 | 1.755 (1.254 , 2.507 ) | 1.40E-03 | 6.66E-01 |
| <45 | 230 | 1.975 (1.330 , 3.026 ) | 1.10E-03 |  | 2.126 (1.499 , 3.084 ) | 3.76E-05 |  |
| **Gender** |  |  |  |  |  |  |  |
| Male | 289 | 1.544 (1.142 , 2.121 ) | 5.86E-03 | 9.66E-01 | 1.851 (1.366 , 2.550 ) | 1.04E-04 | 4.63E-01 |
| Female | 165 | 1.465 (1.011 , 2.169 ) | 4.80E-02 |  | 1.931 (1.283 , 2.993 ) | 2.20E-03 |  |
| **Smoking** |  |  |  |  |  |  |  |
| No | 369 | 1.479 (1.140 , 1.939 ) | 3.75E-03 | 4.02E-01 | 1.851 (1.424 , 2.433 ) | 6.10E-06 | 6.78E-01 |
| Yes | 85 | 1.174 (0.613 , 2.323 ) | 6.30E-01 |  | 1.608 (0.903 , 2.971 ) | 1.13E-01 |  |
| **Drinking** |  |  |  |  |  |  |  |
| No | 381 | 1.447 (1.123 , 1.881 ) | 4.90E-03 | 8.29E-01 | 1.833 (1.419 , 2.393 ) | 5.02E-06 | 4.91E-01 |
| Yes | 73 | 1.960 (0.746 , 6.880 ) | 2.24E-01 |  | 1.680 (0.840 , 3.632 ) | 1.56E-01 |  |
| **Diabetes** |  |  |  |  |  |  |  |
| No | 283 | 1.495 (1.076 , 2.108 ) | 1.87E-02 | 4.70E-01 | 1.857 (1.363 , 2.575 ) | 1.25E-04 | 8.42E-01 |
| Yes | 171 | 1.510 (1.018 , 2.306 ) | 4.66E-02 |  | 1.918 (1.266 , 2.985 ) | 2.72E-03 |  |
| **Hypertension** |  |  |  |  |  |  |  |
| No | 309 | 1.315 (1.006 , 1.740 ) | 4.90E-02 | 3.09E-01 | 1.728 (1.305 , 2.317 ) | 1.76E-04 | 9.71E-01 |
| Yes | 145 | 1.996 (1.217 , 3.417 ) | 8.11E-03 |  | 2.022 (1.240 , 3.413 ) | 6.05E-03 |  |
| **CKD** |  |  |  |  |  |  |  |
| No | 338 | 1.799 (1.333 , 2.472 ) | 1.83E-04 | 3.54E-01 | 2.016 (1.504 , 2.751 ) | 4.91E-06 | 3.21E-01 |
| Yes | 116 | 1.070 (0.676 , 1.713 ) | 7.74E-01 |  | 1.450 (0.894 , 2.408 ) | 1.38E-01 |  |
| **CLD** |  |  |  |  |  |  |  |
| No | 381 | 1.383 (1.072 , 1.801 ) | 1.39E-02 | 3.58E-01 | 1.817 (1.386 , 2.408 ) | 2.11E-05 | 5.68E-01 |
| Yes | 84 | 1.841 (1.008 , 3.590 ) | 5.61E-02 |  | 1.544 (0.812 , 3.040 ) | 1.89E-01 |  |
| Analyses adjusted for age, gender, BMI (for TyG and CTI), smoking, alcohol consumption, CKD, CLD, family history of CVD, HR, Hb, ALT, AST, BUN, and Cr, except for the variable used as the grouping factor. | | | | | | | |

| **Table S7 Subgroup analysis of the associations of TyG and TyG-BMI with CVD in patients with AD in the NHANES** | | | | | | | |
| --- | --- | --- | --- | --- | --- | --- | --- |
| **Classification** |  | **TyG** | | | **TyG-BMI** | | |
|  | **N** | **OR (95%CI)** | **P.value** | **Interaction P** | **OR (95%CI)** | **P.value** | **Interaction P** |
| **Age** |  |  |  |  |  |  |  |
| ≥45 | 218 | 1.614 (1.055 , 2.468 ) | 2.86E-02 | 8.74E-01 | 1.559 (1.091 , 2.229 ) | 1.75E-02 | 6.08E-01 |
| <45 | 268 | 1.742 (1.124 , 2.701 ) | 1.47E-02 |  | 1.659 (1.048 , 2.626 ) | 3.21E-02 |  |
| **Gender** |  |  |  |  |  |  |  |
| Male | 198 | 1.433 (0.935 , 2.197 ) | 9.34E-02 | 6.39E-02 | 1.566 (1.042 , 2.352 ) | 3.27E-02 | 7.27E-01 |
| Female | 288 | 2.160 (1.331 , 3.506 ) | 3.13E-03 |  | 1.634 (1.145 , 2.333 ) | 8.32E-03 |  |
| **Smoking** |  |  |  |  |  |  |  |
| No | 344 | 1.498 (1.064 , 2.108 ) | 2.26E-02 | 3.20E-01 | 1.406 (1.067 , 1.854 ) | 1.76E-02 | 9.10E-02 |
| Yes | 142 | 3.458 (0.887 , 13.480 ) | 6.92E-02 |  | 2.389 (1.148 , 4.971 ) | 2.26E-02 |  |
| **Drinking** |  |  |  |  |  |  |  |
| No | 152 | 1.847 (0.914 , 3.732 ) | 8.29E-02 | 8.17E-01 | 1.676 (1.108 , 2.536 ) | 1.67E-02 | 6.20E-01 |
| Yes | 334 | 1.534 (1.012 , 2.324 ) | 4.42E-02 |  | 1.558 (1.164 , 2.086 ) | 4.03E-03 |  |
| **Hypertension** |  |  |  |  |  |  |  |
| No | 377 | 1.645 (1.128 , 2.398 ) | 1.16E-02 | 9.36E-01 | 1.768 (1.216 , 2.570 ) | 3.97E-03 | 2.57E-01 |
| Yes | 109 | 1.673 (0.722 , 3.876 ) | 1.46E-01 |  | 1.207 (0.697 , 2.090 ) | 3.55E-01 |  |
| Analyses adjusted for age, gender, race, BMI (for TyG and CTI), smoking, alcohol consumption, CKD, CLD, family history of CVD, HR, Hb, ALT, AST, BUN, and Cr, except for the variable used as the grouping factor. | | | | | | | |

| **Table S8 Subgroup analysis of the associations of CTI and CTI-BMI with CVD in patients with AD in the real-world** | | | | | | | |
| --- | --- | --- | --- | --- | --- | --- | --- |
| **Classification** |  | **CTI** | | | **CTI-BMI** | | |
|  | **N** | **OR (95%CI)** | **P.value** | **Interaction P** | **OR (95%CI)** | **P.value** | **Interaction P** |
| **Age** |  |  |  |  |  |  |  |
| ≥45 | 224 | 1.580 (1.152 , 2.208 ) | 5.60E-03 | 5.61E-01 | 1.991 (1.411 , 2.877 ) | 1.42E-04 | 4.97E-01 |
| <45 | 230 | 1.881 (1.315 , 2.758 ) | 7.61E-04 |  | 2.237 (1.568 , 3.272 ) | 1.60E-05 |  |
| **Gender** |  |  |  |  |  |  |  |
| Male | 289 | 1.717 (1.291 , 2.317 ) | 2.78E-04 | 7.81E-01 | 2.094 (1.533 , 2.920 ) | 6.31E-06 | 4.58E-01 |
| Female | 165 | 2.162 (1.376 , 3.556 ) | 1.34E-03 |  | 2.138 (1.405 , 3.375 ) | 6.29E-04 |  |
| **Smoking** |  |  |  |  |  |  |  |
| No | 369 | 1.786 (1.383 , 2.336 ) | 1.37E-05 | 3.20E-01 | 2.071 (1.587 , 2.742 ) | 1.60E-07 | 5.16E-01 |
| Yes | 85 | 1.234 (0.660 , 2.292 ) | 5.02E-01 |  | 1.592 (0.880 , 2.971 ) | 1.29E-01 |  |
| **Drinking** |  |  |  |  |  |  |  |
| No | 381 | 1.709 (1.337 , 2.208 ) | 2.69E-05 | 8.80E-01 | 2.025 (1.565 , 2.655 ) | 1.55E-07 | 4.20E-01 |
| Yes | 73 | 2.054 (0.932 , 5.298 ) | 9.78E-02 |  | 1.759 (0.845 , 4.038 ) | 1.48E-01 |  |
| **Diabetes** |  |  |  |  |  |  |  |
| No | 283 | 1.669 (1.230 , 2.305 ) | 1.34E-03 | 2.21E-01 | 2.018 (1.478 , 2.814 ) | 1.74E-05 | 6.38E-01 |
| Yes | 171 | 2.164 (1.418 , 3.442 ) | 5.94E-04 |  | 2.413 (1.554 , 3.905 ) | 1.62E-04 |  |
| **Hypertension** |  |  |  |  |  |  |  |
| No | 309 | 1.537 (1.172 , 2.040 ) | 2.27E-03 | 2.29E-01 | 1.917 (1.444 , 2.588 ) | 1.13E-05 | 9.52E-01 |
| Yes | 145 | 2.615 (1.541 , 4.685 ) | 6.41E-04 |  | 2.309 (1.397 , 3.990 ) | 1.63E-03 |  |
| **CKD** |  |  |  |  |  |  |  |
| No | 338 | 2.041 (1.507 , 2.821 ) | 7.70E-06 | 5.77E-01 | 2.219 (1.648 , 3.051 ) | 3.53E-07 | 3.49E-01 |
| Yes | 116 | 1.334 (0.823 , 2.208 ) | 2.49E-01 |  | 1.643 (1.009 , 2.757 ) | 5.08E-02 |  |
| **CLD** |  |  |  |  |  |  |  |
| No | 381 | 1.650 (1.273 , 2.164 ) | 2.08E-04 | 7.22E-01 | 2.029 (1.543 , 2.707 ) | 7.23E-07 | 7.86E-01 |
| Yes | 84 | 1.813 (0.972 , 3.567 ) | 6.84E-02 |  | 1.572 (0.829 , 3.068 ) | 1.68E-01 |  |
| Analyses adjusted for age, gender, BMI (for TyG and CTI), smoking, alcohol consumption, CKD, CLD, family history of CVD, HR, Hb, ALT, AST, BUN, and Cr, except for the variable used as the grouping factor. | | | | | | | |

| **Table S9 Subgroup analysis of the associations of CTI and CTI-BMI with CVD in patients with AD in the NHANES** | | | | | | | |
| --- | --- | --- | --- | --- | --- | --- | --- |
| **Classification** |  | **CTI** | | | **CTI-BMI** | | |
|  | **N** | **OR (95%CI)** | **P.value** | **Interaction P** | **OR (95%CI)** | **P.value** | **Interaction P** |
| **Age** |  |  |  |  |  |  |  |
| ≥45 | 218 | 1.515 (1.016 , 2.260 ) | 4.22E-02 | 3.06E-01 | 1.511 (1.052 , 2.169 ) | 2.75E-02 | 8.27E-01 |
| <45 | 268 | 2.500 (1.357 , 4.609 ) | 4.97E-03 |  | 1.763 (1.112 , 2.796 ) | 1.80E-02 |  |
| **Gender** |  |  |  |  |  |  |  |
| Male | 198 | 1.851 (1.104 , 3.102 ) | 2.22E-02 | 9.55E-01 | 1.592 (1.072 , 2.364 ) | 2.36E-02 | 9.05E-01 |
| Female | 288 | 1.702 (1.044 , 2.773 ) | 3.41E-02 |  | 1.436 (0.927 , 2.226 ) | 1.01E-01 |  |
| **Smoking** |  |  |  |  |  |  |  |
| No | 344 | 1.514 (1.064 , 2.155 ) | 2.31E-02 | 9.77E-02 | 1.385 (1.044 , 1.837 ) | 2.56E-02 | 7.29E-02 |
| Yes | 142 | 3.205 (1.457 , 7.053 ) | 6.28E-03 |  | 2.608 (1.187 , 5.732 ) | 1.99E-02 |  |
| **Drinking** |  |  |  |  |  |  |  |
| No | 152 | 1.705 (0.860 , 3.379 ) | 1.18E-01 | 9.43E-01 | 1.642 (1.101 , 2.449 ) | 1.73E-02 | 6.39E-01 |
| Yes | 334 | 1.766 (1.142 , 2.729 ) | 1.26E-02 |  | 1.422 (1.008 , 2.007 ) | 4.55E-02 |  |
| **Hypertension** |  |  |  |  |  |  |  |
| No | 377 | 1.766 (1.142 , 2.729 ) | 1.26E-02 | 2.35E-01 | 1.422 (1.008 , 2.007 ) | 4.55E-02 | 1.91E-01 |
| Yes | 109 | 1.510 (0.659 , 3.463 ) | 2.12E-01 |  | 1.193 (0.685 , 2.080 ) | 3.86E-01 |  |
| Analyses adjusted for age, gender, race, BMI (for TyG and CTI), smoking, alcohol consumption, CKD, CLD, family history of CVD, HR, Hb, ALT, AST, BUN, and Cr, except for the variable used as the grouping factor. | | | | | | | |

| **Table S10 Sensitivity analysis after truncating the upper and lower 5% of extreme values in the real-world** | | | | | | |
| --- | --- | --- | --- | --- | --- | --- |
| **Models** | **Per 1 SD increase** | **P.value** | **Q2 vs Q1** | **P.value** | **Q3 vs Q1** | **P.value** |
| **TyG** |  |  |  |  |  |  |
| Model 1 | 1.274 (1.041 , 1.562 ) | 1.91E-02 | 1.584 (0.958 , 2.638 ) | 7.47E-02 | 1.682 (1.019 , 2.796 ) | 4.31E-02 |
| Model 2 | 1.338 (1.079 , 1.667 ) | 8.60E-03 | 1.799 (1.056 , 3.095 ) | 3.19E-02 | 1.848 (1.086 , 3.173 ) | 2.45E-02 |
| Model 3 | 1.327 (1.063 , 1.664 ) | 1.32E-02 | 1.845 (1.071 , 3.212 ) | 2.86E-02 | 1.831 (1.065 , 3.177 ) | 2.97E-02 |
| **TyG-BMI** |  |  |  |  |  |  |
| Model 1 | 1.312 (1.074 , 1.611 ) | 8.43E-03 | 2.629 (1.558 , 4.517 ) | 3.58E-04 | 2.707 (1.605 , 4.647 ) | 2.32E-04 |
| Model 2 | 1.608 (1.285 , 2.025 ) | 3.97E-05 | 2.994 (1.724 , 5.302 ) | 1.25E-04 | 3.738 (2.130 , 6.710 ) | 6.34E-06 |
| Model 3 | 1.714 (1.352 , 2.188 ) | 1.08E-05 | 3.178 (1.804 , 5.718 ) | 8.20E-05 | 4.364 (2.414 , 8.100 ) | 1.74E-06 |
| **CTI** |  |  |  |  |  |  |
| Model 1 | 1.558 (1.264 , 1.934 ) | 4.21E-05 | 2.119 (1.265 , 3.597 ) | 4.77E-03 | 2.382 (1.427 , 4.034 ) | 1.03E-03 |
| Model 2 | 1.586 (1.271 , 1.993 ) | 5.71E-05 | 2.062 (1.199 , 3.593 ) | 9.60E-03 | 2.418 (1.413 , 4.199 ) | 1.45E-03 |
| Model 3 | 1.578 (1.259 , 1.992 ) | 9.47E-05 | 2.141 (1.235 , 3.764 ) | 7.28E-03 | 2.355 (1.364 , 4.125 ) | 2.35E-03 |
| **CTI-BMI** |  |  |  |  |  |  |
| Model 1 | 1.473 (1.201 , 1.819 ) | 2.46E-04 | 2.637 (1.545 , 4.588 ) | 4.60E-04 | 3.419 (2.018 , 5.923 ) | 7.20E-06 |
| Model 2 | 1.770 (1.412 , 2.238 ) | 1.10E-06 | 2.778 (1.586 , 4.966 ) | 4.35E-04 | 4.544 (2.575 , 8.238 ) | 3.13E-07 |
| Model 3 | 1.896 (1.494 , 2.432 ) | 2.44E-07 | 2.993 (1.682 , 5.447 ) | 2.46E-04 | 5.197 (2.868 , 9.713 ) | 1.10E-07 |
| Model 1: unadjusted. Model 2: analyzed adjusted for age, gender, smoking and drinking. Model 3: analyzed adjusted for age, gender, BMI (TyG and CTI), smoking, drinking, CKD, CLD, family history of CVD, HR, HB, ALT, AST, BUN, Cr. | | | | | | |

| **Table S11 Sensitivity analysis after truncating the upper and lower 5% of extreme values in the NHANES** | | | | | | |
| --- | --- | --- | --- | --- | --- | --- |
| **Models** | **Per 1 SD increase** | **P.value** | **Q2 vs Q1** | **P.value** | **Q3 vs Q1** | **P.value** |
| **TyG** |  |  |  |  |  |  |
| Model 1 | 1.780 (1.355 , 2.339 ) | 1.18E-04 | 1.536 (0.619 , 3.814 ) | 3.46E-01 | 2.849 (1.549 , 5.241 ) | 1.27E-03 |
| Model 2 | 1.752 (1.346 , 2.281 ) | 1.24E-04 | 1.457 (0.516 , 4.111 ) | 4.66E-01 | 2.631 (1.405 , 4.925 ) | 3.53E-03 |
| Model 3 | 1.692 (1.231 , 2.325 ) | 2.11E-03 | 1.355 (0.450 , 4.081 ) | 5.77E-01 | 2.315 (1.150 , 4.662 ) | 2.05E-02 |
| **TyG-BMI** |  |  |  |  |  |  |
| Model 1 | 1.543 (1.218 , 1.954 ) | 6.42E-04 | 3.829 (1.257 , 11.664 ) | 1.94E-02 | 6.256 (2.165 , 18.079 ) | 1.20E-03 |
| Model 2 | 1.593 (1.236 , 2.053 ) | 6.94E-04 | 3.989 (1.163 , 13.684 ) | 2.90E-02 | 7.559 (2.407 , 23.739 ) | 1.02E-03 |
| Model 3 | 1.543 (1.179 , 2.018 ) | 2.61E-03 | 3.695 (1.105 , 12.352 ) | 3.49E-02 | 6.473 (1.957 , 21.413 ) | 3.47E-03 |
| **CTI** |  |  |  |  |  |  |
| Model 1 | 1.758 (1.263 , 2.446 ) | 1.34E-03 | 3.256 (1.305 , 8.121 ) | 1.27E-02 | 4.956 (2.228 , 11.026 ) | 2.37E-04 |
| Model 2 | 1.774 (1.248 , 2.521 ) | 2.17E-03 | 3.545 (1.422 , 8.838 ) | 8.05E-03 | 4.901 (2.078 , 11.561 ) | 6.33E-04 |
| Model 3 | 1.726 (1.222 , 2.438 ) | 3.08E-03 | 3.629 (1.265 , 10.412 ) | 1.84E-02 | 4.698 (1.797 , 12.283 ) | 2.70E-03 |
| **CTI-BMI** |  |  |  |  |  |  |
| Model 1 | 1.473 (1.142 , 1.900 ) | 3.73E-03 | 3.742 (1.158 , 12.091 ) | 2.84E-02 | 5.940 (1.895 , 18.619 ) | 3.10E-03 |
| Model 2 | 1.550 (1.179 , 2.037 ) | 2.54E-03 | 4.140 (1.178 , 14.542 ) | 2.79E-02 | 6.941 (2.085 , 23.108 ) | 2.44E-03 |
| Model 3 | 1.491 (1.118 , 1.989 ) | 8.30E-03 | 4.088 (1.179 , 14.173 ) | 2.79E-02 | 6.225 (1.802 , 21.504 ) | 5.38E-03 |
| Model 1: unadjusted. Model 2: analyzed adjusted for age, gender, race, smoking and drinking. Model 3: analyzed adjusted for age, gender, race, BMI (TyG and CTI), smoking, drinking, CKD, CLD, family history of CVD, HR, HB, ALT, AST, BUN, Cr. | | | | | | |

| **Table S12 Sensitivity analysis after imputing missing values by multiple imputation in the real-world** | | | | | | |
| --- | --- | --- | --- | --- | --- | --- |
| **Models** | **Per 1 SD increase** | **P.value** | **Q2 vs Q1** | **P.value** | **Q3 vs Q1** | **P.value** |
| **TyG** |  |  |  |  |  |  |
| Model 1 | 1.254 (1.046 , 1.507 ) | 1.49E-02 | 1.521 (0.971 , 2.395 ) | 6.83E-02 | 1.613 (1.030 , 2.539 ) | 3.73E-02 |
| Model 2 | 1.321 (1.093 , 1.602 ) | 4.23E-03 | 1.670 (1.048 , 2.678 ) | 3.20E-02 | 1.807 (1.136 , 2.893 ) | 1.30E-02 |
| Model 3 | 1.500 (1.199 , 1.888 ) | 4.48E-04 | 2.049 (1.200 , 3.530 ) | 9.01E-03 | 2.253 (1.354 , 3.789 ) | 1.94E-03 |
| **TyG-BMI** |  |  |  |  |  |  |
| Model 1 | 1.225 (1.022 , 1.472 ) | 2.90E-02 | 1.918 (1.212 , 3.059 ) | 5.76E-03 | 2.084 (1.319 , 3.321 ) | 1.81E-03 |
| Model 2 | 1.411 (1.160 , 1.725 ) | 6.65E-04 | 2.466 (1.528 , 4.027 ) | 2.55E-04 | 2.674 (1.635 , 4.434 ) | 1.08E-04 |
| Model 3 | 1.480 (1.183 , 1.861 ) | 6.82E-04 | 2.518 (1.495 , 4.303 ) | 6.02E-04 | 2.871 (1.648 , 5.089 ) | 2.40E-04 |
| **CTI** |  |  |  |  |  |  |
| Model 1 | 1.391 (1.157 , 1.680 ) | 5.14E-04 | 1.814 (1.148 , 2.889 ) | 1.13E-02 | 2.070 (1.313 , 3.291 ) | 1.88E-03 |
| Model 2 | 1.461 (1.205 , 1.779 ) | 1.32E-04 | 2.042 (1.270 , 3.314 ) | 3.46E-03 | 2.122 (1.328 , 3.424 ) | 1.82E-03 |
| Model 3 | 1.665 (1.330 , 2.101 ) | 1.16E-05 | 2.244 (1.338 , 3.801 ) | 2.37E-03 | 2.616 (1.522 , 4.555 ) | 5.72E-04 |
| **CTI-BMI** |  |  |  |  |  |  |
| Model 1 | 1.250 (1.042 , 1.502 ) | 1.68E-02 | 1.821 (1.154 , 2.894 ) | 1.04E-02 | 1.849 (1.173 , 2.935 ) | 8.54E-03 |
| Model 2 | 1.444 (1.185 , 1.767 ) | 3.06E-04 | 2.125 (1.322 , 3.451 ) | 2.03E-03 | 2.449 (1.508 , 4.025 ) | 3.41E-04 |
| Model 3 | 1.536 (1.228 , 1.932 ) | 1.97E-04 | 2.240 (1.333 , 3.811 ) | 2.57E-03 | 2.726 (1.575 , 4.797 ) | 4.07E-04 |
| Model 1: unadjusted. Model 2: analyzed adjusted for age, gender, smoking and drinking. Model 3: analyzed adjusted for age, gender, BMI (TyG and CTI), smoking, drinking, family history of CVD, HR, HB, ALT, AST, BUN, Cr. | | | | | | |

| **Table S13 Sensitivity analysis after imputing missing values by multiple imputation in the NHANES** | | | | | | |
| --- | --- | --- | --- | --- | --- | --- |
| **Models** | **Per 1 SD increase** | **P.value** | **Q2 vs Q1** | **P.value** | **Q3 vs Q1** | **P.value** |
| **TyG** |  |  |  |  |  |  |
| Model 1 | 1.553 (1.189 , 2.029 ) | 1.87E-03 | 0.995 (0.440 , 2.253 ) | 9.91E-01 | 1.871 (0.986 , 3.548 ) | 5.48E-02 |
| Model 2 | 1.533 (1.165 , 2.016 ) | 3.14E-03 | 0.963 (0.398 , 2.329 ) | 9.32E-01 | 1.583 (0.849 , 2.953 ) | 1.44E-01 |
| Model 3 | 1.507 (1.061 , 2.141 ) | 2.36E-02 | 0.878 (0.338 , 2.276 ) | 7.80E-01 | 1.396 (0.691 , 2.821 ) | 3.39E-01 |
| **TyG-BMI** |  |  |  |  |  |  |
| Model 1 | 1.449 (1.148 , 1.829 ) | 2.52E-03 | 4.305 (1.547 , 11.981 ) | 6.34E-03 | 6.161 (2.134 , 17.792 ) | 1.29E-03 |
| Model 2 | 1.587 (1.207 , 2.087 ) | 1.54E-03 | 3.677 (1.265 , 10.694 ) | 1.82E-02 | 6.638 (2.327 , 18.940 ) | 7.86E-04 |
| Model 3 | 1.761 (1.326 , 2.340 ) | 3.49E-04 | 4.402 (1.342 , 14.448 ) | 1.65E-02 | 8.878 (2.845 , 27.700 ) | 5.40E-04 |
| **CTI** |  |  |  |  |  |  |
| Model 1 | 1.691 (1.282 , 2.230 ) | 4.34E-04 | 3.646 (1.592 , 8.351 ) | 3.07E-03 | 4.011 (1.734 , 9.280 ) | 1.80E-03 |
| Model 2 | 1.746 (1.286 , 2.372 ) | 7.08E-04 | 2.841 (1.307 , 6.173 ) | 9.75E-03 | 3.872 (1.895 , 7.914 ) | 4.69E-04 |
| Model 3 | 1.573 (1.118 , 2.213 ) | 1.12E-02 | 2.624 (1.128 , 6.101 ) | 2.66E-02 | 3.055 (1.377 , 6.779 ) | 7.86E-03 |
| **CTI-BMI** |  |  |  |  |  |  |
| Model 1 | 1.434 (1.140 , 1.803 ) | 2.87E-03 | 7.644 (2.274 , 25.696 ) | 1.60E-03 | 8.138 (2.312 , 28.649 ) | 1.71E-03 |
| Model 2 | 1.585 (1.206 , 2.084 ) | 1.56E-03 | 5.635 (1.545 , 20.555 ) | 1.02E-02 | 8.156 (2.272 , 29.275 ) | 1.99E-03 |
| Model 3 | 1.717 (1.286 , 2.292 ) | 6.71E-04 | 6.400 (1.649 , 24.838 ) | 9.21E-03 | 9.508 (2.411 , 37.500 ) | 2.34E-03 |
| Model 1: unadjusted. Model 2: analyzed adjusted for age, gender, smoking and drinking. Model 3: analyzed adjusted for age, gender, race, BMI (TyG and CTI), smoking, drinking, family history of CVD, HR, HB, ALT, AST, BUN, Cr. | | | | | | |

| **Table S14 Sensitivity analyses by adding different covariates to the base model in the real-world dataset** | | | | | | |
| --- | --- | --- | --- | --- | --- | --- |
| **Models** | **Per 1 SD increase** | **P.value** | **Q2 vs Q1** | **P.value** | **Q3 vs Q1** | **P.value** |
| **Adjusted for AD severity in addition to the base model** | | | | | | |
| **TyG** | 1.459 (1.168 , 1.838 ) | 1.05E-03 | 2.254 (1.310 , 3.930 ) | 3.67E-03 | 2.235 (1.299 , 3.891 ) | 3.98E-03 |
| **TyG-BMI** | 1.808 (1.429 , 2.308 ) | 1.20E-06 | 3.274 (1.860 , 5.894 ) | 5.33E-05 | 4.791 (2.663 , 8.860 ) | 3.02E-07 |
| **CTI** | 1.693 (1.349 , 2.146 ) | 8.23E-06 | 2.149 (1.245 , 3.760 ) | 6.52E-03 | 2.754 (1.599 , 4.820 ) | 3.09E-04 |
| **CTI-BMI** | 1.986 (1.566 , 2.545 ) | 2.80E-08 | 3.073 (1.734 , 5.571 ) | 1.56E-04 | 5.432 (3.019 , 10.081 ) | 3.49E-08 |
| **Adjusted for Glucose-lowering and Lipid-lowering in addition to the base model** | | | | | | |
| **TyG** | 1.443 (1.152 , 1.821 ) | 1.66E-03 | 2.189 (1.275 , 3.805 ) | 4.88E-03 | 2.134 (1.237 , 3.723 ) | 6.87E-03 |
| **TyG-BMI** | 1.793 (1.416 , 2.289 ) | 1.78E-06 | 3.182 (1.811 , 5.713 ) | 7.50E-05 | 4.851 (2.695 , 8.977 ) | 2.52E-07 |
| **CTI** | 1.676 (1.334 , 2.124 ) | 1.33E-05 | 2.092 (1.212 , 3.658 ) | 8.63E-03 | 2.652 (1.538 , 4.648 ) | 5.31E-04 |
| **CTI-BMI** | 1.976 (1.557 , 2.535 ) | 3.91E-08 | 2.996 (1.694 , 5.420 ) | 2.08E-04 | 5.450 (3.025 , 10.121 ) | 3.44E-08 |
| **Adjusted for diabetes, hypertension, and lipid-related variables in addition to the base model** | | | | | | |
| **TyG** | 1.734 (1.348 , 2.254 ) | 2.54E-05 | 2.528 (1.458 , 4.449 ) | 1.09E-03 | 2.746 (1.544 , 4.955 ) | 6.68E-04 |
| **TyG-BMI** | 1.897 (1.487 , 2.444 ) | 4.06E-07 | 3.376 (1.902 , 6.135 ) | 4.45E-05 | 5.291 (2.901 , 9.949 ) | 1.09E-07 |
| **CTI** | 1.966 (1.534 , 2.549 ) | 1.68E-07 | 2.443 (1.396 , 4.340 ) | 1.97E-03 | 3.521 (1.975 , 6.396 ) | 2.58E-05 |
| **CTI-BMI** | 2.077 (1.627 , 2.685 ) | 9.75E-09 | 3.118 (1.747 , 5.696 ) | 1.54E-04 | 5.935 (3.252 , 11.199 ) | 1.51E-08 |
| Base model: adjusted for age, gender, BMI (TyG and CTI), smoking, drinking, CKD,CLD, family history of CVD, HR, HB, ALT, AST, BUN, Cr. | | | | | | |

| **Table S15 Sensitivity analyses by adding different covariates to the base model in the NHANES** | | | | | | |
| --- | --- | --- | --- | --- | --- | --- |
| **Models** | **Per 1 SD increase** | **P.value** | **Q2 vs Q1** | **P.value** | **Q3 vs Q1** | **P.value** |
| **Adjusted for Glucose-lowering and Lipid-lowering in addition to the base model** | | | | | | |
| **TyG** | 1.712 (1.172 , 2.501 ) | 7.22E-03 | 1.405 (0.485 , 4.066 ) | 5.16E-01 | 2.209 (0.937 , 5.207 ) | 6.85E-02 |
| **TyG-BMI** | 1.552 (1.133 , 2.126 ) | 7.96E-03 | 2.855 (0.817 , 9.975 ) | 9.65E-02 | 5.064 (1.483 , 17.292 ) | 1.17E-02 |
| **CTI** | 1.790 (1.192 , 2.686 ) | 6.72E-03 | 2.904 (0.972 , 8.676 ) | 5.58E-02 | 4.574 (1.597 , 13.101 ) | 6.40E-03 |
| **CTI-BMI** | 1.542 (1.122 , 2.120 ) | 9.53E-03 | 3.331 (0.950 , 11.676 ) | 5.93E-02 | 5.142 (1.540 , 17.166 ) | 9.76E-03 |
| **Adjusted for diabetes, hypertension, and lipid-related variables in addition to the base model** | | | | | | |
| **TyG** | 1.538 (1.055 , 2.240 ) | 2.67E-02 | 1.234 (0.407 , 3.747 ) | 6.99E-01 | 1.607 (0.720 , 3.587 ) | 2.35E-01 |
| **TyG-BMI** | 1.443 (1.081 , 1.925 ) | 1.47E-02 | 3.560 (1.027 , 12.342 ) | 4.56E-02 | 5.668 (1.615 , 19.884 ) | 8.70E-03 |
| **CTI** | 1.631 (1.147 , 2.319 ) | 8.33E-03 | 3.047 (1.094 , 8.486 ) | 3.42E-02 | 4.219 (1.661 , 10.714 ) | 3.89E-03 |
| **CTI-BMI** | 1.439 (1.083 , 1.913 ) | 1.42E-02 | 3.818 (1.020 , 14.290 ) | 4.69E-02 | 5.641 (1.731 , 18.385 ) | 5.81E-03 |
| Base model: adjusted for age, gender, BMI (TyG and CTI), smoking, CKD,CLD, drinking, family history of CVD, HR, HB, ALT, AST, BUN, Cr. | | | | | | |

| **Table S16 Sensitivity analysis excluding BMI adjustment** | | | | | | |
| --- | --- | --- | --- | --- | --- | --- |
| **Models** | **Per 1 SD increase** | **P.value** | **Q2 vs Q1** | **P.value** | **Q3 vs Q1** | **P.value** |
| **NHANES** | | | | | | |
| **TyG** | 1.549 (1.101 , 2.179 ) | 1.38E-02 | 1.264 (0.416 , 3.841 ) | 6.69E-01 | 1.910 (0.910 , 4.009 ) | 8.45E-02 |
| **CTI** | 1.589 (1.086 , 2.324 ) | 1.90E-02 | 3.369 (1.136 , 9.992 ) | 2.99E-02 | 3.686 (1.245 , 10.912 ) | 2.03E-02 |
| **Real-world** | | | | | | |
| **TyG** | 1.430 (1.137 , 1.812 ) | 2.56E-03 | 2.234 (1.296 , 3.902 ) | 4.16E-03 | 2.053 (1.177 , 3.620 ) | 1.19E-02 |
| **CTI** | 1.669 (1.323 , 2.123 ) | 2.09E-05 | 2.012 (1.155 , 3.549 ) | 1.44E-02 | 2.533 (1.454 , 4.476 ) | 1.16E-03 |
| Adjusted for age, gender, smoking, drinking, CKD,CLD, family history of CVD, HR, HB, ALT, AST, BUN, Cr. | | | | | | |

| **Table S17 Sensitivity analyses by excluding atrial fibrillation and cardiac death in the real-world dataset** | | | | | | |
| --- | --- | --- | --- | --- | --- | --- |
| **Models** | **Per 1 SD increase** | **P.value** | **Q2 vs Q1** | **P.value** | **Q3 vs Q1** | **P.value** |
| **TyG** |  |  |  |  |  |  |
| Model 1 | 1.337 (1.088 , 1.650 ) | 6.14E-03 | 1.711 (1.025 , 2.884 ) | 4.12E-02 | 1.855 (1.116 , 3.115 ) | 1.81E-02 |
| Model 2 | 1.407 (1.132 , 1.761 ) | 2.36E-03 | 1.882 (1.101 , 3.252 ) | 2.18E-02 | 2.019 (1.182 , 3.489 ) | 1.08E-02 |
| Model 3 | 1.555 (1.207 , 2.021 ) | 7.55E-04 | 2.117 (1.212 , 3.748 ) | 9.06E-03 | 2.209 (1.233 , 4.004 ) | 8.22E-03 |
| **TyG-BMI** |  |  |  |  |  |  |
| Model 1 | 1.378 (1.124 , 1.696 ) | 2.18E-03 | 3.017 (1.677 , 5.572 ) | 2.97E-04 | 4.804 (2.633 , 9.051 ) | 5.79E-07 |
| Model 2 | 1.660 (1.326 , 2.093 ) | 1.25E-05 | 3.151 (1.792 , 5.677 ) | 9.11E-05 | 4.223 (2.385 , 7.688 ) | 1.32E-06 |
| Model 3 | 1.770 (1.384 , 2.282 ) | 7.19E-06 | 3.279 (1.824 , 6.043 ) | 9.69E-05 | 4.684 (2.543 , 8.894 ) | 1.25E-06 |
| **CTI** |  |  |  |  |  |  |
| Model 1 | 1.595 (1.288 , 1.991 ) | 2.52E-05 | 2.127 (1.256 , 3.660 ) | 5.51E-03 | 2.537 (1.509 , 4.343 ) | 5.39E-04 |
| Model 2 | 1.637 (1.309 , 2.065 ) | 2.18E-05 | 2.051 (1.184 , 3.603 ) | 1.12E-02 | 2.565 (1.492 , 4.486 ) | 7.71E-04 |
| Model 3 | 1.810 (1.410 , 2.348 ) | 4.77E-06 | 2.309 (1.305 , 4.152 ) | 4.47E-03 | 2.963 (1.646 , 5.429 ) | 3.49E-04 |
| **CTI-BMI** |  |  |  |  |  |  |
| Model 1 | 1.537 (1.250 , 1.904 ) | 5.87E-05 | 3.014 (1.734 , 5.379 ) | 1.26E-04 | 3.790 (2.197 , 6.730 ) | 2.84E-06 |
| Model 2 | 1.821 (1.452 , 2.305 ) | 3.53E-07 | 3.017 (1.677 , 5.572 ) | 2.97E-04 | 4.804 (2.633 , 9.051 ) | 5.79E-07 |
| Model 3 | 1.952 (1.526 , 2.525 ) | 1.80E-07 | 3.188 (1.759 , 5.936 ) | 1.77E-04 | 5.246 (2.843 , 10.022 ) | 2.31E-07 |
| Model 1: unadjusted. Model 2: analyzed adjusted for age, gender, smoking and drinking. Model 3: analyzed adjusted for age, gender, BMI (TyG and CTI), smoking, drinking,, family history of CVD, HR, HB, ALT, AST, BUN, Cr. | | | | | | |

| **Table S18 Comparison of AUC values across different models** | | | | | | |
| --- | --- | --- | --- | --- | --- | --- |
| **Feature** | **Model 1** | **Model 2** | **AUC 1** | **AUC 2** | **ΔAUC** | **P value** |
| **Base+TyG** | **LR** | **KNN** | 0.517 | 0.493 | 0.023 | 4.18E-01 |
|  | **LR** | **SVC** | 0.517 | 0.638 | -0.121 | 5.72E-04 |
|  | **LR** | **MLP** | 0.517 | 0.616 | -0.099 | 5.05E-04 |
|  | **LR** | **XGBoost** | 0.517 | 0.633 | -0.116 | 6.65E-04 |
|  | **LR** | **DT** | 0.517 | 0.546 | -0.029 | 4.19E-01 |
|  | **LR** | **RF** | 0.517 | 0.604 | -0.087 | 3.97E-03 |
|  | **KNN** | **SVC** | 0.493 | 0.638 | -0.144 | 4.00E-05 |
|  | **KNN** | **MLP** | 0.493 | 0.616 | -0.122 | 3.19E-04 |
|  | **KNN** | **XGBoost** | 0.493 | 0.633 | -0.140 | 1.57E-04 |
|  | **KNN** | **DT** | 0.493 | 0.546 | -0.052 | 1.78E-01 |
|  | **KNN** | **RF** | 0.493 | 0.604 | -0.111 | 4.29E-03 |
|  | **SVC** | **MLP** | 0.638 | 0.616 | 0.022 | 4.65E-01 |
|  | **SVC** | **XGBoost** | 0.638 | 0.633 | 0.004 | 8.98E-01 |
|  | **SVC** | **DT** | 0.638 | 0.546 | 0.092 | 6.06E-03 |
|  | **SVC** | **RF** | 0.638 | 0.604 | 0.033 | 2.86E-01 |
|  | **MLP** | **XGBoost** | 0.616 | 0.633 | -0.017 | 5.66E-01 |
|  | **MLP** | **DT** | 0.616 | 0.546 | 0.070 | 4.21E-02 |
|  | **MLP** | **RF** | 0.616 | 0.604 | 0.011 | 6.96E-01 |
|  | **XGBoost** | **DT** | 0.633 | 0.546 | 0.087 | 2.62E-03 |
|  | **XGBoost** | **RF** | 0.633 | 0.604 | 0.029 | 1.99E-01 |
|  | **DT** | **RF** | 0.546 | 0.604 | -0.058 | 1.52E-02 |
| **Base+TyG-BMI** | **LR** | **KNN** | 0.524 | 0.512 | 0.012 | 6.76E-01 |
|  | **LR** | **SVC** | 0.524 | 0.616 | -0.091 | 2.33E-02 |
|  | **LR** | **MLP** | 0.524 | 0.650 | -0.126 | 7.32E-04 |
|  | **LR** | **XGBoost** | 0.524 | 0.666 | -0.142 | 8.96E-05 |
|  | **LR** | **DT** | 0.524 | 0.585 | -0.061 | 1.12E-01 |
|  | **LR** | **RF** | 0.524 | 0.657 | -0.132 | 2.87E-05 |
|  | **KNN** | **SVC** | 0.512 | 0.616 | -0.104 | 5.03E-03 |
|  | **KNN** | **MLP** | 0.512 | 0.650 | -0.138 | 2.97E-04 |
|  | **KNN** | **XGBoost** | 0.512 | 0.666 | -0.154 | 1.77E-05 |
|  | **KNN** | **DT** | 0.512 | 0.585 | -0.073 | 4.71E-02 |
|  | **KNN** | **RF** | 0.512 | 0.657 | -0.145 | 8.42E-05 |
|  | **SVC** | **MLP** | 0.616 | 0.650 | -0.034 | 3.16E-01 |
|  | **SVC** | **XGBoost** | 0.616 | 0.666 | -0.051 | 1.79E-01 |
|  | **SVC** | **DT** | 0.616 | 0.585 | 0.030 | 4.10E-01 |
|  | **SVC** | **RF** | 0.616 | 0.657 | -0.041 | 2.26E-01 |
|  | **MLP** | **XGBoost** | 0.650 | 0.666 | -0.016 | 6.21E-01 |
|  | **MLP** | **DT** | 0.650 | 0.585 | 0.065 | 7.32E-02 |
|  | **MLP** | **RF** | 0.650 | 0.657 | -0.007 | 8.24E-01 |
|  | **XGBoost** | **DT** | 0.666 | 0.585 | 0.081 | 1.43E-03 |
|  | **XGBoost** | **RF** | 0.666 | 0.657 | 0.010 | 6.59E-01 |
|  | **DT** | **RF** | 0.585 | 0.657 | -0.071 | 2.36E-03 |
| **Base+CTI** | **LR** | **KNN** | 0.533 | 0.478 | 0.055 | 5.19E-02 |
|  | **LR** | **SVC** | 0.533 | 0.623 | -0.090 | 2.61E-02 |
|  | **LR** | **MLP** | 0.533 | 0.656 | -0.123 | 5.94E-04 |
|  | **LR** | **XGBoost** | 0.533 | 0.634 | -0.101 | 3.53E-03 |
|  | **LR** | **DT** | 0.533 | 0.560 | -0.027 | 4.58E-01 |
|  | **LR** | **RF** | 0.533 | 0.628 | -0.096 | 2.41E-03 |
|  | **KNN** | **SVC** | 0.478 | 0.623 | -0.144 | 2.01E-04 |
|  | **KNN** | **MLP** | 0.478 | 0.656 | -0.177 | 1.93E-06 |
|  | **KNN** | **XGBoost** | 0.478 | 0.634 | -0.156 | 1.54E-05 |
|  | **KNN** | **DT** | 0.478 | 0.560 | -0.082 | 3.36E-02 |
|  | **KNN** | **RF** | 0.478 | 0.628 | -0.150 | 8.93E-05 |
|  | **SVC** | **MLP** | 0.623 | 0.656 | -0.033 | 3.42E-01 |
|  | **SVC** | **XGBoost** | 0.623 | 0.634 | -0.011 | 7.59E-01 |
|  | **SVC** | **DT** | 0.623 | 0.560 | 0.063 | 6.46E-02 |
|  | **SVC** | **RF** | 0.623 | 0.628 | -0.006 | 8.49E-01 |
|  | **MLP** | **XGBoost** | 0.656 | 0.634 | 0.022 | 4.95E-01 |
|  | **MLP** | **DT** | 0.656 | 0.560 | 0.096 | 4.92E-03 |
|  | **MLP** | **RF** | 0.656 | 0.628 | 0.027 | 3.31E-01 |
|  | **XGBoost** | **DT** | 0.634 | 0.560 | 0.074 | 7.93E-03 |
|  | **XGBoost** | **RF** | 0.634 | 0.628 | 0.005 | 8.27E-01 |
|  | **DT** | **RF** | 0.560 | 0.628 | -0.068 | 8.66E-03 |
| **Base+CTI-BMI** | **LR** | **KNN** | 0.527 | 0.519 | 0.008 | 7.86E-01 |
|  | **LR** | **SVC** | 0.527 | 0.614 | -0.087 | 3.03E-02 |
|  | **LR** | **MLP** | 0.527 | 0.645 | -0.118 | 1.34E-03 |
|  | **LR** | **XGBoost** | 0.527 | 0.653 | -0.127 | 7.03E-04 |
|  | **LR** | **DT** | 0.527 | 0.511 | 0.015 | 7.03E-01 |
|  | **LR** | **RF** | 0.527 | 0.650 | -0.123 | 1.04E-04 |
|  | **KNN** | **SVC** | 0.519 | 0.614 | -0.095 | 1.22E-02 |
|  | **KNN** | **MLP** | 0.519 | 0.645 | -0.126 | 7.51E-04 |
|  | **KNN** | **XGBoost** | 0.519 | 0.653 | -0.135 | 2.17E-04 |
|  | **KNN** | **DT** | 0.519 | 0.511 | 0.007 | 8.41E-01 |
|  | **KNN** | **RF** | 0.519 | 0.650 | -0.131 | 6.06E-04 |
|  | **SVC** | **MLP** | 0.614 | 0.645 | -0.031 | 3.73E-01 |
|  | **SVC** | **XGBoost** | 0.614 | 0.653 | -0.040 | 2.88E-01 |
|  | **SVC** | **DT** | 0.614 | 0.511 | 0.102 | 9.66E-03 |
|  | **SVC** | **RF** | 0.614 | 0.650 | -0.036 | 2.65E-01 |
|  | **MLP** | **XGBoost** | 0.645 | 0.653 | -0.009 | 8.00E-01 |
|  | **MLP** | **DT** | 0.645 | 0.511 | 0.133 | 1.06E-03 |
|  | **MLP** | **RF** | 0.645 | 0.650 | -0.005 | 8.57E-01 |
|  | **XGBoost** | **DT** | 0.653 | 0.511 | 0.142 | 8.04E-06 |
|  | **XGBoost** | **RF** | 0.653 | 0.650 | 0.004 | 8.88E-01 |
|  | **DT** | **RF** | 0.511 | 0.650 | -0.139 | 3.19E-05 |
|  |  |  |  |  |  |  |

| **Table S19 Evaluation of the effectiveness of different machine learning models in the real-world** | | | | | |
| --- | --- | --- | --- | --- | --- |
| **Index** | **ML model** | **Precision** | **Recall** | **F1-score** | **Accuracy** |
| **Base** | **LR** | 0.423 | 0.372 | 0.346 | 0.551 |
|  | **KNN** | 0.402 | 0.503 | 0.377 | 0.469 |
|  | **SVC** | 0.421 | 0.993 | 0.485 | 0.326 |
|  | **MLP** | 0.433 | 0.055 | 0.095 | 0.663 |
|  | **XGboost** | 0.622 | 0.083 | 0.143 | 0.683 |
|  | **DT** | 0.454 | 0.910 | 0.510 | 0.441 |
|  | **RF** | 0.455 | 0.855 | 0.502 | 0.458 |
|  |  |  |  |  |  |
| **Base + TyG** | **LR** | 0.432 | 0.683 | 0.447 | 0.460 |
|  | **KNN** | 0.400 | 0.490 | 0.372 | 0.471 |
|  | **SVC** | 0.420 | 1.000 | 0.485 | 0.322 |
|  | **MLP** | 0.608 | 0.214 | 0.301 | 0.683 |
|  | **XGboost** | 0.648 | 0.117 | 0.193 | 0.687 |
|  | **DT** | 0.457 | 0.703 | 0.473 | 0.500 |
|  | **RF** | 0.454 | 0.924 | 0.512 | 0.438 |
|  |  |  |  |  |  |
| **Base + TyG-BMI** | **LR** | 0.467 | 0.379 | 0.373 | 0.593 |
|  | **KNN** | 0.425 | 0.510 | 0.397 | 0.504 |
|  | **SVC** | 0.423 | 1.000 | 0.488 | 0.330 |
|  | **MLP** | 0.641 | 0.228 | 0.320 | 0.692 |
|  | **XGboost** | 0.767 | 0.552 | 0.102 | 0.689 |
|  | **DT** | 0.468 | 0.655 | 0.471 | 0.531 |
|  | **RF** | 0.496 | 0.772 | 0.523 | 0.551 |
|  |  |  |  |  |  |
| **Base + CTI** | **LR** | 0.439 | 0.579 | 0.427 | 0.504 |
|  | **KNN** | 0.395 | 0.497 | 0.370 | 0.460 |
|  | **SVC** | 0.422 | 1.000 | 0.487 | 0.328 |
|  | **MLP** | 0.614 | 0.379 | 0.437 | 0.687 |
|  | **XGboost** | 0.747 | 0.076 | 0.136 | 0.692 |
|  | **DT** | 0.478 | 0.855 | 0.524 | 0.504 |
|  | **RF** | 0.474 | 0.897 | 0.527 | 0.487 |
|  |  |  |  |  |  |
| **Base + CTI-BMI** | **LR** | 0.438 | 0.497 | 0.402 | 0.529 |
|  | **KNN** | 0.421 | 0.483 | 0.386 | 0.509 |
|  | **SVC** | 0.421 | 1.000 | 0.486 | 0.324 |
|  | **MLP** | 0.634 | 0.524 | 0.603 | 0.694 |
|  | **XGboost** | 0.814 | 0.645 | 0.658 | 0.687 |
|  | **DT** | 0.509 | 0.717 | 0.521 | 0.579 |
|  | **RF** | 0.507 | 0.752 | 0.528 | 0.570 |
|  |  |  |  |  |  |
